# Superoxide Dismutase Multigene Family from a Primitive Chondrostean Sturgeon, *Acipenser baerii*: Molecular Characterization, Evolution, and Antioxidant Defense during Development and Pathogen Infection

**DOI:** 10.3390/antiox10020232

**Published:** 2021-02-03

**Authors:** Chan-Hee Kim, Eun Jeong Kim, Yoon Kwon Nam

**Affiliations:** Department of Marine Bio-Materials and Aquaculture, College of Fisheries Sciences, Pukyong National University, 45 Yongso-ro, Nam-gu, Busan 48513, Korea; chkim@pknu.ac.kr (C.-H.K.); kejung03@naver.com (E.J.K.)

**Keywords:** superoxide dismutase multigene family, antioxidant defense, phylogeny, development, immune response, *Acipenser baerii*

## Abstract

Three distinct superoxide dismutases (SODs)—copper/zinc-SOD (SOD1), manganese-SOD (SOD2), and extracellular copper/zinc-SOD (SOD3)—were identified from a primitive chondrostean fish, *Acipenser baerii*, enabling the comparison of their transcriptional regulation patterns during development, prelarval ontogeny, and immune stimulation. Each *A. baerii* SOD isoform (AbSOD) shared conserved structural features with its vertebrate orthologs; however, phylogenetic analyses hypothesized a different evolutionary history for AbSOD3 relative to AbSOD1 and AbSOD2 in the vertebrate lineage. The AbSOD isoforms showed different tissue distribution patterns; AbSOD1 was predominantly expressed in most tissues. The expression of the AbSOD isoforms showed isoform-dependent dynamic modulation according to embryonic development and prelarval ontogenic behaviors. Prelarval microinjections revealed that lipopolysaccharide only induced AbSOD3 expression, while *Aeromonas hydrophila* induced the expression of AbSOD2 and AbSOD3. In fingerlings, the transcriptional response of each AbSOD isoform to bacterial infection was highly tissue-specific, and the three isoforms exhibited different response patterns within a given tissue type; AbSOD3 was induced the most sensitively, and its induction was the most pronounced in the kidneys and skin. Collectively, these findings suggest isoform-dependent roles for the multigene SOD family in antioxidant defenses against the oxidative stress associated with development and immune responses in these endangered sturgeon fish.

## 1. Introduction

Aerobic respiration, indispensable in aerobic organisms, gives rise to the constant generation of reactive oxygen species (ROS) as a byproduct, formed by the partial reduction of oxygen [1]. Excessive ROS can overwhelm cells, damaging biological molecules and leading to irreversible cell damage and/or eventual death [2,3]. However, ROS in the proper locations and concentrations activate many signaling pathways, facilitating the actions of growth factors, cytokines, and calcium [3,4]. Given their dual role as a destructive and constructive entity, aerobic organisms have evolved systems to delicately balance ROS production and scavenging [1,5]; however, this balance is readily disturbed by various stresses that significantly increase cellular ROS concentrations, called pro-oxidant factors [6,7]. The molecular mechanisms underlying the delicate regulation of ROS usually involve enzymes, such as antioxidant enzymes (AOEs). The superoxide dismutases (SODs; EC 1.15.1.1), regarded as the first line of defense in the AOE system, are a family of redox-active metalloenzymes that catalyze the dismutation of superoxide radicals to molecular oxygen and hydrogen peroxide [8,9]. It has been demonstrated that different SOD isoforms have different evolutionary histories in the animal kingdom, as evidenced by homology and phylogenetic data [10,11,12].

Fish are the most diverse aquatic vertebrates and live in virtually all aquatic habitats [13,14]. Their physiology is directly affected by adverse changes in ambient environments regarding temperature, oxygen, pollutants, and pathogenic infections, which can act as stress factors [7,15]. Moreover, fish are highly vulnerable to environmental changes in early life. Embryonic and larval development require dynamic cellular activities with high oxygen/energy demands, involving changes in metabolism, physiology, and body architecture that can cause large variations in ROS production [16,17,18]. In the context of our understanding of their evolutionary history and distinct physiological characteristics, fish, like all other aerobic organisms, possess AOE systems to protect against ROS-driven oxidative damage, and their AOE systems, including SODs, have been attracting attention [7,19]. Fish employ three different SOD isoforms that are characterized by their metal cofactors and cellular localization: copper/zinc SOD, present in the cytosol (Cu/Zn-SOD; SOD1); manganese SOD, located within mitochondria (Mn-SOD; SOD2); and extracellular Cu/Zn-SOD, predominantly localized within the extracellular matrices of tissues (EC-SOD; SOD3) [9,20,21]. Several biochemical and molecular studies have shown that fish SODs are modulated by their lifecycles, including embryonic and larval development, as well as exposure to environmental stresses, including endotoxins and pathogenic infections, suggesting that fish SODs play crucial roles not only in the development but also in the maintenance of a homeostatic balance between the innate immune response and antioxidant defenses [22,23,24,25,26,27,28,29,30,31,32,33]. However, most studies on fish SODs have been limited to enzymatic measurements, which represent total or pooled SOD activity without distinguishing among the three SOD isoforms, and to transcriptional modulation, which is only representative of one or two distinct fish SODs. Such observations do not allow a deeper insight into the isoform-specific and/or isoform-dependent roles of fish SODs [8,34]. A comparison between the different fish SOD isoforms would be valuable for investigating the different and coordinated mechanisms by which the distinct isoforms protect against oxidative stress.

Chondrostean sturgeons (Acipenseriformes) are of the basal and ancient lineage of Actinopterygii (ray-finned fish), often referred to as “living fossils” [35]. Due to their unique phylogenetic position, sturgeons are considered a useful model for studying the evolution of biological systems, including the diversification of host defense mechanisms in the vertebrate lineage [13,36,37]. Sturgeon have also long been considered a valuable fisheries product (as a source of caviar); however, due to their endangered status in nature, commercial sturgeon products must be produced aquaculturally [38]. Due to this requirement, the development of effective and efficient protocol(s) for the production of artificially propagated sturgeon seedlings is critical [39,40,41,42]. A comprehensive understanding of developmentally regulated genes that are involved in embryonic and/or larval protection could be a fundamental basis for a better understanding of the inherent capabilities of sturgeon to conduct embryogenesis and ontogenesis in response to variable environmental factors [43]. However, despite their importance, the roles of antioxidant SODs in the development of sturgeon have not been extensively studied. To date, only a couple of studies have reported changes in SOD activities during early life [31,44], but no comparative molecular expression data for the three SOD isoforms are available. More importantly, with the expansion of sturgeon aquaculture over the past few decades, a parallel increase in infectious diseases in recent years has become an important issue in sturgeon farming [45,46,47]. Although some efforts to characterize the host defense process against microbial pathogens in sturgeon species have been made or are ongoing, the regulation of SOD isoforms during infection has not yet been studied. There exists a large body of information regarding mammalian and teleostean SODs, but the roles of SODs in innate immunity in primitive chondrostean fish have been little studied.

The objectives of this study were (1) to characterize the genetic determinants of SOD1 (Cu/Zn-SOD), SOD2 (Mn-SOD), and SOD3 (EC-Cu/Zn-SOD) from Siberian sturgeon *Acipenser baerii*, including an analysis of their evolutionary relationships with vertebrate orthologs based on reconstructed molecular phylogenetic trees; (2) to examine the gene expression patterns of the three isoforms in tissues, embryos, and prelarvae from a comparative perspective; and (3) to scrutinize the isoform-dependent patterns of the transcriptional responses of SODs to immunostimulatory challenges with lipopolysaccharide (LPS) endotoxins and *Aeromonas hydrophila* in prelarvae and fingerlings.

## 2. Materials and Methods

### 2.1. Experimental Animals and Ethics Declaration

The Siberian sturgeon *A. baerii* specimens used in this study were obtained from 11 stages of embryonic development, 6 prelarval ontogenic stages, and a fingerling stage and were artificially propagated at the Experimental Fish Culture Station (EFCS), Pukyong National University (PKNU), Busan, South Korea. The preparation of the experimental specimens and the stages of embryonic and ontogenic development are described in the following sections. The fish were handled and treated according to the guidelines of the National Act on Laboratory Animals and as approved by the Animal Care and Use Committee of PKNU (approval number 201818).

### 2.2. Molecular Cloning of SOD cDNAs

From our local next-generation sequencing (NGS) database generated with adult *A. baerii* female liver transcriptomes, partial NGS clones showing significant homology to vertebrate SOD orthologs were selected based on a BLASTx search against the non-redundant (NR) protein database (DB) of NCBI GenBank. For each SOD isoform, the NGS clones were assembled into a contig using Sequencher (Gene Codes, Ann Arbor, MI, USA) and used as a template for rapid amplification of cDNA ends (RACE) experiments for both 5′ and 3′ ends. RACE cloning was carried out with a commercial kit (Thermo Fisher Scientific, Waltham, MA, USA) according to the manufacturer’s instructions. The continuous cDNA sequence of each SOD isoform containing a full-length open reading frame (ORF) region was produced from the liver total RNA of the fingerlings using reverse transcription–PCR (RT-PCR) amplification and TA cloned into the pGEM-T easy vector (Promega, Madison, WI, USA). At least six recombinant RT-PCR clones for each SOD isoform were sequenced in both directions to obtain the representative cDNA sequence of each isoform. The oligonucleotide primers used are listed in Table S1.

### 2.3. In Silico Sequence Analysis and Structural Modeling

The three SOD-isoform cDNAs isolated from *A. baerii* were denoted as AbSOD1, AbSOD2, and AbSOD3, respectively, based on the BLAST homology search results. The full-length nucleotide sequences for AbSOD1, AbSOD2, and AbSOD3 are available under GenBank accession numbers MW032449, MW032450, and MW032451, respectively. For each AbSOD cDNA sequence, the putative ORF region and corresponding protein sequence were deduced using the ORF Finder [48]. The molecular weight (Mw; kDa), theoretical isoelectric point (*p*I), and instability index (II) values of each AbSOD isoform were determined using the online ProtParam tool [49]. The presence and locations of the mitochondrial transfer peptide (MTP) and signal peptide (SP) sequences of the SOD proteins were predicted using the TargetP-2.0 server [50], and the potential subcellular localization was analyzed using CELLO v.2. [51]. *N*-glycosylation sites in the AbSODs were identified using NetNGlyc [52]. The domain framework and motif sequences for the AbSOD isoforms were identified using InterProScan [53].

A three dimensional (3D) structure was built for each AbSOD isoform by homology structure modeling using the Phyre2 server [54]. The quality of the predicted structural models of the AbSOD proteins was assessed with the SWISS-MODEL structure assessment server [55], and ligand clustering and binding site prediction for the structural models were further performed using the 3DLigandSite server [56]. PyMoL v1.8.2.0 (http://www.pymol.org) was used to visualize the structural models [57] and potential ligand-binding sites and for the superimposition of the predicted AbSOD models with corresponding human SOD structures.

### 2.4. Molecular Phylogeny and Multiple Sequence Alignment

The evolutionary relationship and genetic affiliation of each AbSOD with its potential orthologs were assessed by the reconstruction of molecular phylogenetic trees. Orthologous sequences for each AbSOD isoform were searched for by the BLASTp query of each isoform against Actinopterygii (taxid: 7898) in the NCBI NR protein DB, followed by the elimination of partial, hypothetical, and redundant sequences. Representative SOD orthologs from Sarcopterygii (taxid: 8287) including terrestrial vertebrates (tetrapods), coelacanths (Actinistia), and lungfish (Dipnoi) were sampled. The orthologs obtained from species belonging to Chondrichthyes (taxid: 7777) were used to root the inferred tree. MEGA-X was used to determine the best-fit evolutionary model and to infer a phylogenetic tree for each SOD isoform [58]. The maximum likelihood (ML) method based on the determined best-fit evolutionary model was used to construct phylogenetic topologies with 1000 bootstrap replicates for the evaluation of their branch supports. Multiple sequence alignments of each AbSOD isoform along with its representative orthologs sampled from the reconstructed phylogenetic tree were carried out using Clustal Omega [59].

### 2.5. Sampling of Tissues, Embryos, and Prelarvae

To examine the distribution of the AbSOD isoform mRNAs across tissues, 11 somatic tissues including brain, eye, fin (caudal), gill, heart, intestine (mid-intestine), kidney (head kidney), liver, muscle (skeletal of dorsal part), skin (abdominal region), and spleen were surgically removed from eight *A. baerii* fingerling individuals (average total length = 10.1 ± 1.2 cm; average body weight = 3.9 ± 0.6 g). Upon removal, the tissues were immediately frozen in dry ice and stored at −85 °C until use.

To obtain developing embryo samples, we conducted hormone-induced artificial spawning and insemination with three females and three males of mature *A. baerii*. All the procedures, including the selection of mature broodfish, injection of a luteinizing hormone-releasing hormone analog (LHRH; Syndel Laboratories Ltd., Qualicum Beach, BC, Canada), collection of gametes, and artificial fertilization, were performed as described in our previous work [40]. Three embryo batches, each consisting of 20,000 inseminated eggs, were prepared and incubated at 19–20 °C until they hatched [40]. Approximately 50 developing embryos were randomly chosen at the time of fertilization (0 h post fertilization (hpf)), first cleavage (2 hpf), 8 cells (4 hpf), formation of an early blastula (9.5 hpf), onset of gastrulation (19.5 hpf), formation of a small yolk plug (28 hpf), onset of neurulation (33 hpf), formation of an excretory rudiment (37 hpf), formation of a heart rudiment and tail bud (59 hpf), onset of heart beating with S-heart (73 hpf), tail extension to the head (101 hpf), and first hatching (119 hpf). Of the 50 embryos, 10 were used to confirm the developmental stage, and the remaining 40 were frozen and stored at −85 °C. Details of the staging of the embryogenesis in *A. baerii* can be found in our previous studies [40,42]. During development, dead embryos were periodically removed at 8 h intervals, and the dissolved oxygen (DO) levels were maintained at 8.0 ± 0.5 ppm throughout the development.

At mass hatching, the hatchlings were collected within 6 h intervals to obtain synchronized prelarval batches. Approximately 4000 hatched prelarvae were stocked in a rectangular tank (1.2 × 2.0 × 0.4 m (D × W × H)) containing 300 L of 10 μm-filtered groundwater and equipped with a custom-designed filter unit. Three replicate batches were prepared. The DO and temperature were adjusted to 20.0 ± 1.0 °C and 8.0 ± 1.0 ppm, respectively, throughout the prelarval period. The diel photoperiodic cycle was 14:10 h for light (L)/dark (D). The other conditions for the prelarval management are described in our previous work [36,37], and those for the morphological development and differentiation of the prelarvae can be found in [39]. Based on the documentation about the behavioral modifications of *A. baerii* during the prelarval period [60], we monitored the behavioral changes of the prelarvae under the present nursery conditions. During the prelarval period, 30 prelarvae were sampled from each batch at 1, 3, 5, 7, 9, and 11 days post-hatching (dph). From each sampling, 10 fish were microscopically examined to confirm normal ontogenetic development at each age, while the remaining 20 were immediately frozen with dry ice and stored at −85 °C until use.

### 2.6. In Vivo Immunostimulatory Challenges

#### 2.6.1. Microinjection of LPS or Microbial Pathogens into Prelarvae

To examine whether hatched prelarvae were able to modulate AbSOD mRNA expression in response to endotoxins or pathogen infection, which would potentially provoke ROS generation, we microinjected prelarvae with lipopolysaccharides (LPS; *Escherichia coli*; 0111: B4; Sigma-Aldrich, St. Louis, MO, USA) or *Aeromonas hydrophila* (Gram-negative; KCTC 2358); the conditions for the microinjection and anesthesia of prelarvae were as described in our previous work [61]. Briefly, 1-day-old prelarvae (*n* = 48, average body weight (BW) = 14.3 ± 0.42 mg) were anesthetized with 200 ppm of MS-222 (Sigma-Aldrich, USA), which was microinjected along with 20 nL of LPS at 20, 40, and 80 μg/g BW or 1 × 10^3^ CFU/g BW of *A. hydrophila* suspended in phosphate-buffered saline (PBS, pH 7.4) into the border region between the prelarval body and yolk extension. Based on our preliminary examinations, the doses of LPS and *A. hydrophila* used did not cause significant prelarval mortality until 24 hpi; however, about 20–25% cumulative mortality was eventually found in the *A. hydrophila*-injected group at the end of the prelarval stage (10 dph). Each control group for the LPS and *A. hydrophila* microinjections was prepared by using the same number (*n* = 48) of prelarvae microinjected with the same volume of PBS. After the microinjections, the prelarvae were quickly recovered from anesthesia in oxygenated water (>8 ppm of DO). Subsequently, the PBS-injected control and LPS-injected groups were transferred to different net cages (40 × 30 × 25 cm (D × W × H)), all installed together in a large rectangular tank (1.2 × 2.0 × 0.4 m (D × W × H)). The *A. hydrophila-*injected and respective control prelarvae were transferred to two isolated tanks (60 × 40 × 25 cm (D × W × H)) to avoid any potential risks associated with cross infection in the control group. The water temperature and DO levels were maintained at 20.0 ± 1.0 °C and 8.0 ± 1.0 ppm, respectively, during the experimental period. Prelarvae (*n* = 12) were sampled at 6, 12, and 24 hpi from the control and challenged groups, immediately frozen, and stored at −85 °C until use.

#### 2.6.2. *A. hydrophila* Challenge with Fingerlings

To examine the tissue dependence of the pattern of AbSOD expression in response to *A. hydrophila* infection, fingerlings (10.7 ± 0.8 cm; 4.5 ± 0.7 g; *n* = 24) were intraperitoneally injected with 100 μL of *A. hydrophila* at 2 × 10^4^ CFU/g BW, which did not cause mortality until 48 hpi. The control group (*n* = 30) was injected with the same volume of PBS. After injection, the fingerlings were transferred to one of two rectangular tanks (1 × 2 × 0.3 m (D × W × H)) and maintained at 20 °C with 8 ppm of DO. Six individuals were randomly chosen from each tank at 6, 12, 24, and 48 hpi, and four tissues (kidney, liver, skin, and spleen) were individually obtained, frozen, and stored at −85 °C until use.

### 2.7. Nucleic Acid Preparation, RT-qPCR Assay, and Statistics

Total RNA was extracted from tissues, embryos, and whole prelarvae with TRIzol Reagent (Thermo Fisher Scientific) and further purified with the RNeasy Plus Mini Kit (Qiagen, Hilden, Germany), including the DNA elimination step. The integrity of the total RNA was validated by electrophoresis in a 1% MOPS–formaldehyde agarose gel, which was stained with ethidium bromide, and then assessing 28S to 18S rRNA band intensity ratio. The quality and quantity of the total RNA were assessed with a NanoDrop ND-1000 spectrophotometer (Thermo Fisher Scientific, USA) to confirm that both the 260/280 and 260/230 nm ratios were higher than 1.9. An aliquot (2 μg) of total RNA from each sample was reverse-transcribed into cDNA using an Omniscript Reverse Transcription Kit (Qiagen, Germany) using both oligo-d(T)_20_ and random nonamer primers according to the manufacturer’s instructions. The RT products (cDNA) were diluted 10-fold, and 2 μL of the diluted cDNA was used as a template for RT-qPCR amplification. The RT-qPCR was carried out in triplicate using a LightCycler 480 Real-Time PCR System and LightCycler 480 SYBR Green I Master mix (Roche Applied Science, Penzberg, Germany). *A. baerii* 18S rRNA (accession no., AY904463.1) was used as a reference control gene to normalize the AbSOD expression levels across samples [36,37]. The PCR efficiency for each primer pair, including that for the 18S rRNA control, ranged from 0.91 to 1.03. The relative proportions of each AbSOD isoform in each tissue (i.e., tissue distribution assay) or developmental sample (i.e., embryonic and ontogenetic expression assay) were quantified using the 2^−ΔCT^ method [62]. In the immune challenge experiments with prelarvae and fingerlings, the expression levels of AbSOD isoforms in the challenged groups are expressed as fold changes relative to those in the PBS-control group based on the normalization against 18S rRNA using the 2^−ΔΔCT^ method [62]. Differences in expression were assessed using ANOVA, followed by Duncan’s multiple range test and/or Student’s *t*-test, at the level of *p* = 0.05.

## 3. Results

### 3.1. Characteristics of AbSOD cDNAs and Deduced Amino Acid Sequences

**Figure 1:**
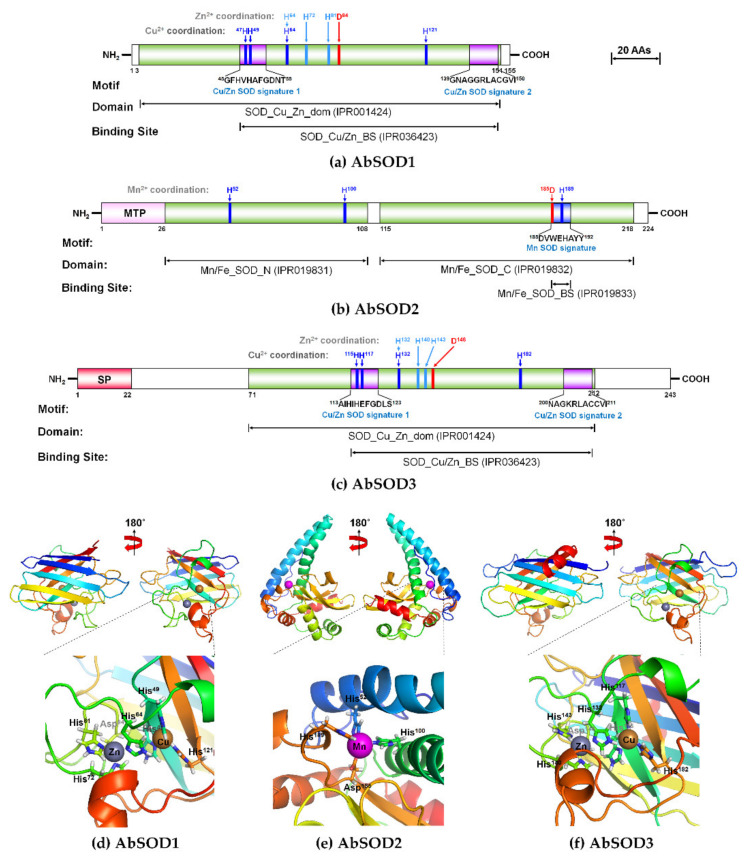
Schematic diagrams showing the predicted protein domain architecture and 3D model for each *Acipenser baerii* SOD (AbSOD) isoform: (**a**) AbSOD1 is composed of a Cu/Zn-SOD domain (147 residues, green) containing a Cu/Zn-SOD binding site (105 residues) with two Cu/Zn-SOD motif signatures. (**b**) The AbSOD2 protein starts with a predicted MTP (26 residues, pink), followed by two Mn/Fe-SOD domains (green) flanked by a central space. The Mn/Fe-SOD binding site (blue) in the Mn/Fe-SOD C-terminal domain is identical to the Mn-SOD signature. (**c**) The AbSOD3 protein comprises a signal peptide (22 residues, red) and a Cu/Zn-SOD domain (141 residues, green) containing a Cu/Zn-SOD binding site (96 residues) with two Cu/Zn-SOD motif signatures. Residues for metal ion coordination are shown at the top of each schematic diagram. (**d**) The AbSOD1 model was constructed from Residues 4 through 154 using human Cu/Zn-SOD (d2c9va1) as the template. (**e**) The AbSOD2 model from Residues 27 to 227 was built using human Mn-SOD (c1n0nB) as a template. (**f**) The AbSOD3 model was built using human extracellular Cu/Zn-SOD (c2jlpA) as a template, with a portion containing the Cu/Zn-SOD domain from Residues 58 to 224. Closer-up views for each model represent the coordination of metal ion(s) with corresponding residues. Cu^2+^ and Zn^2+^ ions in the AbSOD1 and AbSOD3 models are shown in dark gray and brown, respectively, and Mn^2+^ ion in the AbSOD3 model is purple-colored. All models are colored according to the rainbow from N- to C-termini.

The structural schematics of the deduced AbSOD proteins are shown in Figure 1a–c, and the full-length nucleotide sequences and deduced amino acid (aa) sequences are shown in Figures S1–S3. The AbSOD1 cDNA comprised 949 bp, starting with a 5′-untranslated region (UTR) of 122 bp, followed by an ORF of 465 bp and a 3′-UTR of 362 bp including a stop codon (TAA). A putative polyadenylation consensus signal (AATAAA) was located 12 bp upstream of the poly(A^+^) tail (Figure S1). The ORF of AbSOD1 encoded a protein of 155 aa residues with a predicted molecular weight (Mw) of 15.9 kDa, a *p*I of 5.84, and an instability index value of 16.51, regarded as showing it to be stable. In silico protein sequence analysis revealed that the AbSOD1 protein had a conserved Cu/Zn-SOD domain (IPR001424; 4th–151st residues); the Cu/Zn-SOD binding site (IPR036423) was predicted to exist between Residues 45 and 150, containing two Cu/Zn SOD signature consensus patterns (^45^GFHVHAFGDNT^55^ and ^139^GNAGGRLACGVI^150^) (Figure 1a). Meanwhile, MTP and SP sequences were not observed in the AbSOD1 protein, which is consistent with the results of the subcellular localization analysis by CELLO prediction (i.e., the protein is probably located in the cytoplasm, with a 0.789 reliability).

The 1033 bp AbSOD2 cDNA comprised a 5′-UTR of 118 bp, an ORF of 672 bp, and a 3′-UTR of 243 bp containing a stop codon (TAA) and a putative polyadenylation consensus signal (AATAAA) located 12 bp upstream of the poly(A^+^) tail (Figure S2). The AbSOD2 ORF encoded a stable protein (instability index value = 38.9) of 224 aa residues with a Mw of 24.7 kDa and *p*I of 8.29. The AbSOD2 protein was found to be likely to be located in the mitochondria, with a reliability of 2.784, by CELLO prediction, which was confirmed with a strong probability score (0.99) for the presence of the MTP (26 aa residues) at the N-terminus. Additionally, the AbSOD2 protein was predicted to have the following consensus features: (1) two Mn/Fe-SOD domains consisting of Mn/Fe-SOD N-terminal (IPR019831; 27th–108th residues) and Mn/Fe-SOD C-terminal domains (IPR019832; 115th–218th residues); (2) a Mn/Fe-SOD binding site (IPR019833) identical to the Mn-SOD motif signature (^185^DVWEHAYY^192^); and (3) a potential *N*-glycosylation site (^99^NHS^101^) (Figure 1b and Figure S2). The AbSOD3 cDNA was 1923 bp long, consisting of a 5′-UTR of 89 bp, an ORF of 729 bp, and a 3′-UTR of 1105 bp including a TAG stop codon and a putative polyadenylation consensus signal (AATAAA) located 16 bp upstream of the poly(A^+^) tail (Figure S3). The AbSOD3 protein comprised 243 aa residues, with a Mw of 26.5 kDa, *p*I of 9.21, and instability value of 50.54. The signal peptide with the 22-aa sequence in AbSOD3 was identified, and the predicted subcellular localization was extracellular, with a reliability of 1.937. An analysis of the domain framework and motif sequences revealed that the AbSOD3 protein was comparable to the AbSOD1 protein in those regards. Accordingly, AbSOD3 had a conserved Cu/Zn-SOD domain (IPR001424; 71st–212th residues), in which the Cu/Zn-binding site (IPR036423) exhibited 115–211 residues. The binding site included two Cu/Zn-SOD motif signatures (^113^AIHIHEFGDLS^123^ and ^200^NAGKRLACCVI^211^) similar to those of the AbSOD1 protein (Figure 1c). A potential *N*-glycosylation site for the AbSOD3 protein was detected at the Asn^109^ (^109^NQS^111^) residue (Figure S3), indicating that AbSOD3 could become an extracellular glycoprotein through post-translational modification.

The 3D homology models based on the ligand clustering and binding site prediction were constructed on the Phyre2 and 3DLigandSite servers using templates—d2c9va1 (human Cu/Zn-SOD1) for AbSOD1, c1n0nB (human Mn-SOD) for AbSOD2, and c2jlpA (human extracellular Cu/Zn-SOD) for AbSOD (Figure 1d–f). The quality of the 3D models of the three AbSOD proteins was assessed as fairly good (Figure S4). Additionally, superimposing comparisons showed that the overall folds of the structural models of the AbSOD proteins were highly similar to the corresponding X-ray crystallographic structures of human SODs (Figure S5a–c). Notably, the overall folds of the 3D AbSOD1 and AbSOD3 models were similar to each other’s, consistent with the results from the domain framework and motif sequence analyses of the AbSOD1 and AbSOD3 proteins (Figure S5d). Both 3D models mainly contained β-barrel structures composed of eight β-strands and two large loops (Figure 1d,f). The ligand clustering and binding site prediction for the 3D AbSOD1 model demonstrated that the Cu^2+^ ion was coordinated by four His residues (His^47^, His^49^, His^64^, and His^121^), and the Zn^2+^ ion was coordinated by three His residues (His^64^, His^72^, and His^81^) and one Asp residue (Asp^84^) (Figure 1d). Likewise, in the AbSOD3 model, the Cu^2+^ ion was coordinated by four His residues (His^115^, His^117^, His^132^, and His^182^), and the Zn^2+^ ion was coordinated by three His residues (His^132^, His^140^, and His^143^) and one Asp residue (Asp^146^) (Figure 1f). The His^64^ residue in the AbSOD1 protein and the His^132^ residue in the AbSOD3 protein bridged the Cu^2+^ ion to the Zn^2+^ ion. Meanwhile, the AbSOD2 3D model was composed of nine α-helices and three β-strands, where two long α-helices at the N-terminus formed a helical hairpin structure, while the C-terminus contained a three-stranded β-sheet flanked by α-helices on both sides (Figure 1e). In the AbSOD2 3D model, three His residues (His^52^, His^100^, and His^189^) and one Asp residue (Asp^185^) were coordinated with the Mn^2+^ ion at the junction of two long α-helices from the N-terminus and a β-sheet flanked by α-helices from the C-terminus.

### 3.2. Assessment of Evolutionary Relationships and Sequence Homology

A phylogenetic tree for each AbSOD protein was inferred using orthologous protein sequences for 78 SOD1s, 91 SOD2s, and 77 SOD3s from jawed vertebrate groups, including Chondrichthyes, Sarcopterygii, and Actinopterygii. The best-fitting evolutionary models at the protein level were determined to be the WAG + G model for SOD1, JTT + G for SOD2, and JTT + G+I for SOD3.

**Figure 2:**
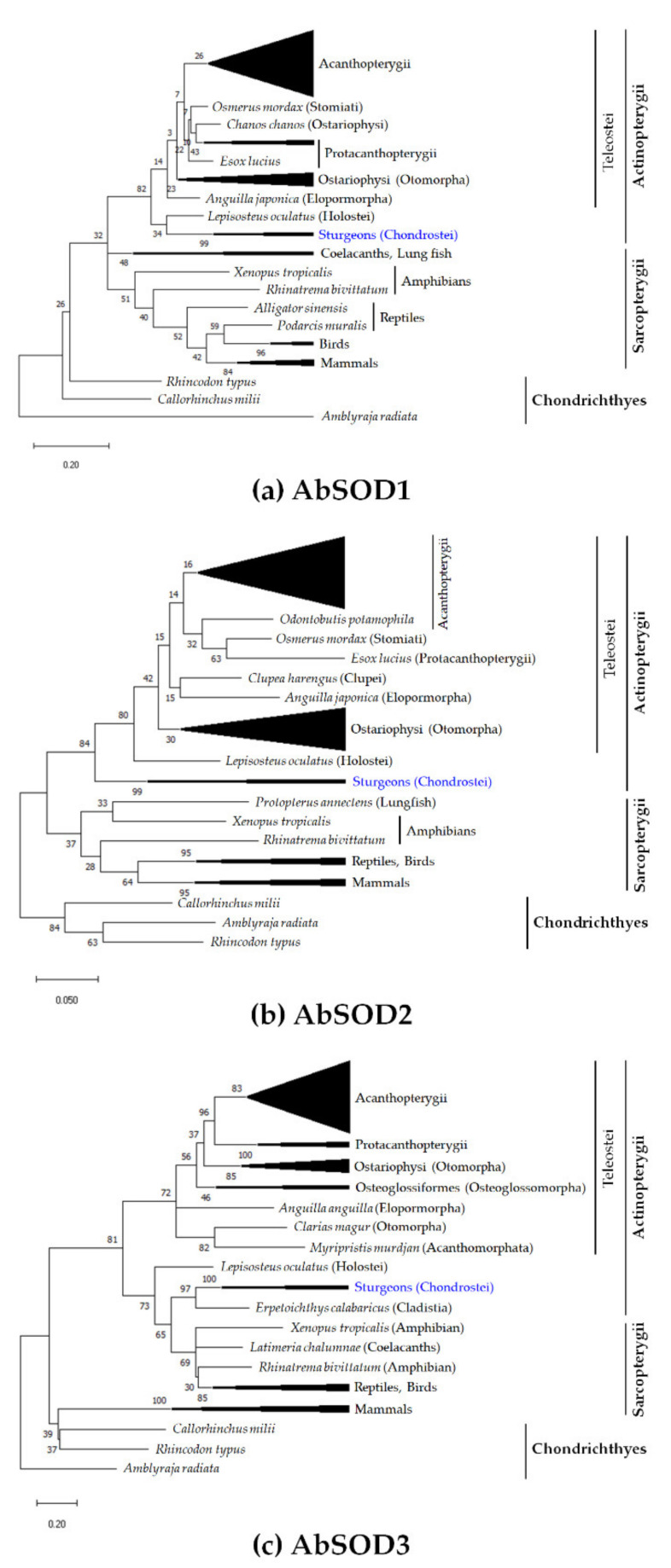
Compressed phylogenetic trees depicting the evolutionary relationships of (**a**) AbSOD1, (**b**) AbSOD2, and (**c**) AbSOD3 proteins with corresponding orthologs from other species in the jawed vertebrate linage. Original trees are shown in Figures S6–S8. Numbers at tree nodes refer to the percent bootstrap values after 1000 replications.

The respective gamma shape values (five rate categories) for AbSOD1, AbSOD2, and AbSOD3 were 0.43, 0.37, and 1.08, with best tree scores (−ln likelihood) of −4996.61, −4610.32, and −8848.61, respectively. In the inferred trees for the SOD1 and SOD2 proteins, the branching topologies were in good agreement with known taxonomic appraisals, an exception being the *Anguilla japonica* (Elopomorpha) SOD2, grouped with *Clupea harengus* (Clupei) SOD2. Although bootstrap support was low for all the branches, we observed that the complied SOD1 and SOD2 sequences appeared to have formed a monophyletic clade of jawed vertebrates with cartilaginous fish rooting. AbSOD1 along with the gar *Lepisosteus oculatus* (Holostei) SOD1 shared a common ancestor with actinopterygian fish, which were a sister clade of sarcopterygian SODs. Consequently, the phylogenetic tree hypothesizes that AbSOD1 occupies a basal evolutionary position of the ray-finned fish (Actinopterygii), from which the teleost SOD1s divergently evolved (Figure 2a and Figure S6). Likewise, AbSOD2 was also placed at a basal position of ray-finned fish (Actinopterygii) that were the sister clade of sarcopterygian SODs (Figure 2b and Figure S7). However, unlike SOD1 and SOD2, the phylogenetic tree of SOD3 showed that the actinopterygian was not resolved in the monophyletic clade, in which AbSOD3 together with known primitive actinopterygian members, including the reedfish *Erpetoichthys calabaricus* (Cladistia) and the gar *L. oculatus* (Holostei), exhibited a closer genetic affiliation with non-mammalian sarcopterygian orthologs in a lobe-finned fish *Latimeria chalumnae* (coelacanth), amphibians, birds, and reptiles than with teleostean orthologs placed in a monophyletic clade. Meanwhile, the mammalian clade was weakly affiliated with two cartilaginous fish SOD3s (*Callorhinchus milli* and *Rhincodon typus*) (Figure 2c and Figure S8).

**Figure 3:**
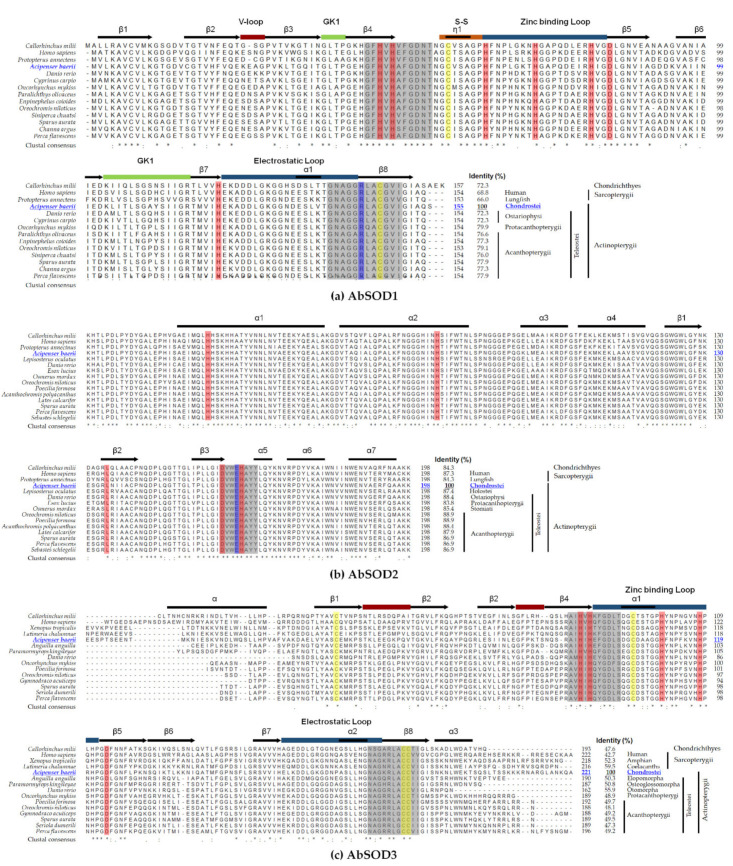
Multiple sequence alignment of (**a**) AbSOD1, (**b**) AbSOD2, and (**c**) AbSOD3 proteins with the selected representative orthologs with respect to the phylogenetic analysis. The predicted motif signature(s) and residues for metal ion(s) coordination are highlighted in gray and red, respectively. Cysteine residues likely to form intramolecular disulfide bond(s) are highlighted in yellow. The illustrating secondary structures at the top of each sequence alignment are shown based on the solved human SOD structure. The symbols at the bottom of the sequence alignments represent the cluster-based consensuses: (*) fully conserved; (:) highly conserved; (.) conserved substitution.

According to multiple sequence alignments, the predicted domain(s) and motif signature(s) from each AbSOD isoform were highly conserved among orthologs, and the predicted residues for metal ion coordination between the AbSOD isoforms and their orthologs were identical (Figure 3). Pairwise comparisons of the AbSODs with corresponding human SOD orthologs, whose structures are well-characterized, were able to facilitate more informative sequence observations [63,64,65]: (1) in AbSOD1, Arg^144^ can be considered an important residue for enzyme activity, and two cysteine residues (Cys^58^ and Cys^147^), forming an intramolecular disulfide bond, are expected to contribute to the stability of the SOD1 protein (Figure 3a); (2) in AbSOD2, Glu^188^ and His^189^ can stabilize oligomerization (Figure 3b); (3) in AbSOD3, four cysteine residues can form two intramolecular disulfide bonds (Cys^65^–Cys^209^ and Cys^126^–Cys^208^) responsible for enzymatic activity (Figure 3c). Estimations of percent sequence homology (Table S2) showed that AbSOD1 was more similar to orthologs from actinopterygian members (74.0–99.4%) than to sarcopterygian fish and tetrapods (51.0–72.7%). The percent sequence homologies were similarly high for most of the SOD2 orthologs examined, ranging from 78.1 to 100%. Meanwhile, it was found that the SOD3 proteins exhibited a relatively wide range of sequence homology across orthologs; AbSOD3 showed 38.5–44.2, 53.1–100, 40.6–50.5, 49.2–56.3, and 38.7–58.0% similarities to orthologs from Chondrichthyes, non-teleost actinopterygians, teleosts, sarcopterygian fish, and sarcopterygian tetrapods, respectively. Collectively, the results from the phylogenetic and homology analyses suggest that each AbSOD isoform exhibits functional orthology to its corresponding ortholog members in the jawed vertebrate lineage.

### 3.3. Tissue Distribution and Relative Expression of A. baerii SOD Isoforms

**Figure 4:**
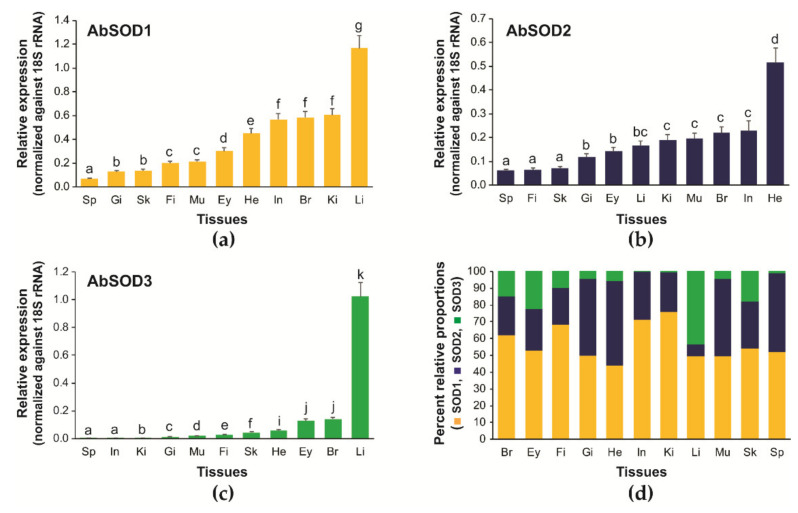
Tissue distribution patterns and basal expression levels of (**a**) AbSOD1, (**b**) AbSOD2, and (**c**) AbSOD3 isoforms as determined by the RT-qPCR assay. Tissue abbreviations represent the brain (Br), eyes (Ey), fins (Fi), gills (Gi), heart (He), intestines (In), kidneys (Ki), liver (Li), muscles (Mu), skin (Sk), and spleen (Sp). For each AbSOD isoform, means ± SDs with different letters (a–k) are significantly different according to ANOVA followed by Duncan’s multiple range test at *p* < 0.05. Along with average expression values, the relative proportions of the three AbSOD isoforms within a given tissue are also indicated in (**d**).

The three AbSOD isoforms were ubiquitously detected in all the tissues examined according to the RT-qPCR assays; however, their basal expression greatly varied across tissues (Figure 4). AbSOD1 showed the highest mRNA expression in the liver, followed by the kidneys, brain, and intestines. Meanwhile, AbSOD1 expression in other tissues was only moderate or weak, which, in descending order of expression, were the heart, eyes, muscles, fin, skin, gills, and spleen (Figure 4a). The expression of AbSOD2 was the highest in the heart, followed by the intestines, brain, muscles, and kidneys. Moderate expression of AbSOD2 was observed in the liver, eyes, and gills, whereas the spleen, fins, and skin exhibited the lowest (Figure 4b). AbSOD3 exhibited the highest expression in the liver. Excluding the predominant expression in the liver, the remaining tissues exhibited only weak expression of AbSOD3 (Figure 4c).

The relative proportional expression of the three AbSOD isoforms differed according to tissue type (Figure 4d). Although that of AbSOD1 was 44.1% in the heart, lower than that of 50.3% for AbSOD2, the AbSOD1 isoform was dominant in other tissue types, ranging from 49.5% in the liver and muscles to 75.8% in the kidneys. The relative proportions of AbSOD2 ranged from 7.1% (liver) to 50.3% (kidneys). The relative abundances of AbSOD3 in the liver (43.4%) and eyes (22.5%) were comparable to those of the AbSOD1 and AbSOD2 isoforms, respectively. However, in other tissues, AbSOD3 showed only minute proportional values, particularly in the intestines, kidneys, and spleen (less than 10%).

### 3.4. Expression Patterns of A. baerii SODs during Embryonic and Ontogenic Development

#### 3.4.1. Embryonic Expression

**Figure 5:**
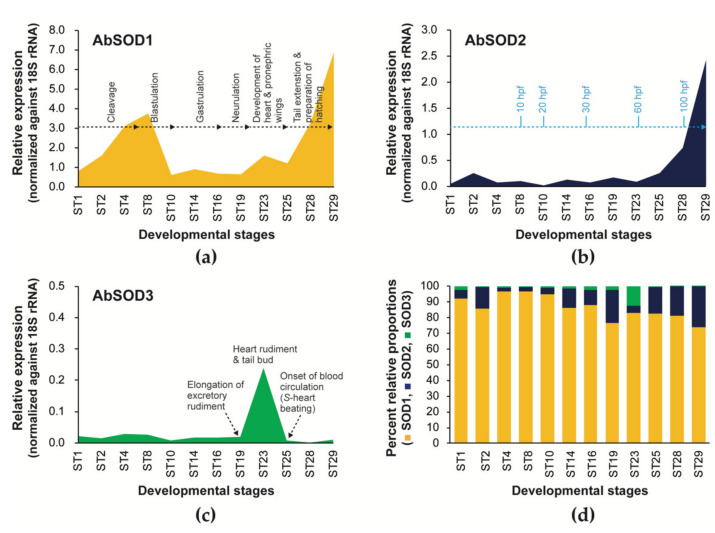
Expression compendium of the isoform-dependent modulation of AbSOD mRNAs in developing *A. baerii* embryos. (**a**) AbSOD1, (**b**) AbSOD2, (**c**) AbSOD3, and (**d**) relative proportions of AbSOD isoforms at given developmental stages. Stage numbers ST1 (just fertilized) to ST29 (hatching) with detailed embryological characteristics of *A. baerii* embryos at each developmental stage refer to those from our previous work [40]. Embryological features in representative stages are summarized in (**a**,**c**), while the time scale in hours post fertilization (hpf) is shown in (**b**). Due to the low expression levels in many developmental intervals (particularly for AbSOD2 and AbSOD3), standard deviations and statistical test results are omitted for clarity. Results of statistical tests with one-way ANOVA followed by Duncan’s multiple range test are provided in Figure S9.

The expression of all three AbSOD isoforms was dynamically modulated during embryonic development (Figure 5). Upon fertilization, the mRNA expression of AbSOD1 was gradually elevated with the progress of early cleavages, peaked at the early blastula stage, and dropped at the onset of gastrulation. Its expression during gastrulation and early neurulation (Stages 14–19) was relatively invariant, while it increased during heart formation and tail budding (Stages 23–25) and remained sharply elevated until the first hatching (Stages 25–29) (Figure 5a). The overall temporal pattern of AbSOD2’s expression was not significantly different from that of AbSOD1; an increase in its mRNA expression began from Stage 23 (the formation of heart rudiment) and continued until hatching. However, the peak that was observed for AbSOD1 during early cleavage and blastula formation was not visualized for the AbSOD2 isoform (Figure 5b). Meanwhile, the temporal expression pattern of AbSOD3 was dissimilar to those of AbSOD1 and AbSOD2; its expression notably increased within a narrow window from Stage 19 (rudimentary excretory wing formed) to Stage 25 (S-heart formation) (Figure 5c). A comparison of the proportional abundances of the three AbSOD isoforms revealed that AbSOD1 was largely dominant throughout embryonic development. The relative abundance of AbSOD2 tended to increase with the progress of development, while that of AbSOD3, however, was very low in most of the stages, except Stage 23.

#### 3.4.2. Ontogenic Expression during Prelarval Period

**Figure 6:**
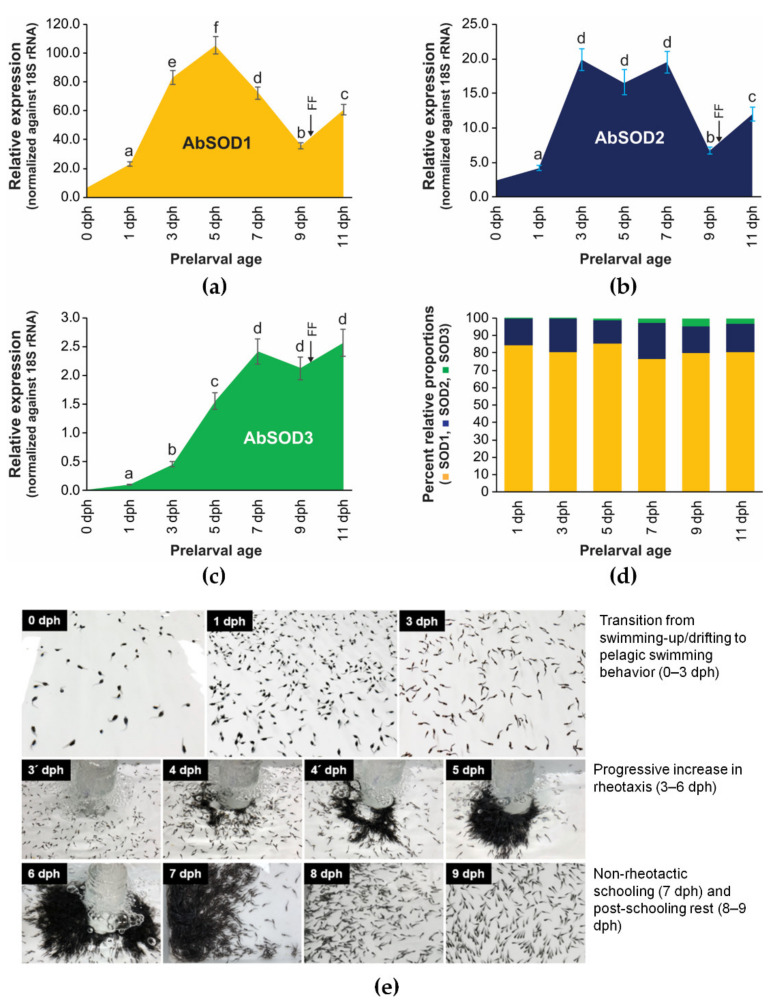
Isoform-dependent modulation patterns of AbSOD mRNAs during the ontogenic development (days post hatching; dph) of *A. baerii* prelarvae. (**a**) AbSOD1, (**b**) AbSOD2, (**c**) AbSOD3, and (**d**) relative proportions of AbSOD isoforms at given prelarval ages. Arrows indicate the first feeding (FF) between 9 and 10 dph. Representative images for behavioral transition patterns during the prelarval period are also provided in (**e**). Morphological differentiations of *A. baerii* during the prelarval period refer to those from our previous work [39], while detailed information on behavioral changes in prelarvae is available in our previous work [66]. In (**e**), images for 3´ and 4´ dph indicate those obtained 6 h after 3 and 4 dph, respectively. For each AbSOD isoform, means ± SDs with different letters (a–f) are significantly different according to ANOVA followed by Duncan’s multiple range test at *p* < 0.05. Expression of each AbSOD isoform at 0 dph was that at Stage 29, shown in Figure 5.

Under the present nursery conditions, the *A. baerii* prelarvae showed behavioral transitions regarding swimming activity, water column preference, rheotaxis, and schooling that were in overall agreement with previously determined patterns. The behavioral transition patterns typically observed in this sturgeon species are addressed in our previous work [66]. During the prelarval period, the mRNA expression of three AbSOD isoforms was vigorously modulated, with isoform-dependent temporal patterns (Figure 6). The expression of AbSOD1 progressively rose from 1 to 5 dph, gradually decreased until 9 dph, and then rebounded at 11 dph (Figure 6a). The elevation of AbSOD2 expression in the initial phase (from 1 to 3 dph) was similar to that of AbSOD1. However, there was no further increase at the subsequently monitored points. Instead, the elevated expression level was not significantly altered until 7 dph, although a small decrease was detectable at 5 dph. At 9 dph, a sharp decrease in AbSOD2 expression was found, followed by a moderate recovery at 11 dph, which was broadly similar to the pattern for AbSOD1 (Figure 6b). Meanwhile, the expression pattern of AbSOD3 was different from those of AbSOD1 and AbSOD2; although there was a small decrease at 9 dph, similar to for the other two isoforms, AbSOD3’s expression was continuously elevated with the ontogenic development of the prelarvae (Figure 6c). A comparison of the proportional abundances of the three AbSOD isoforms showed that AbSOD1 was the dominant isoform throughout the prelarval period, accounting for 77–85% of the total SOD transcript abundance depending on the ontogenic stage. AbSOD2 showed the second highest proportional abundance, ranging from 13 to 21%. The proportion of AbSOD3 expression was very low until 5 dph (0.4–1.3%) and then slightly increased in the later phases (2.6 to 4.8% from 7 to 11 dph) (Figure 6d).

### 3.5. Transcriptional Responses of A. baerii SODs to Immunostimulatory Treatments

#### 3.5.1. Expression in Prelarvae Microinjected with LPS or *A. hydrophila*

**Figure 7:**
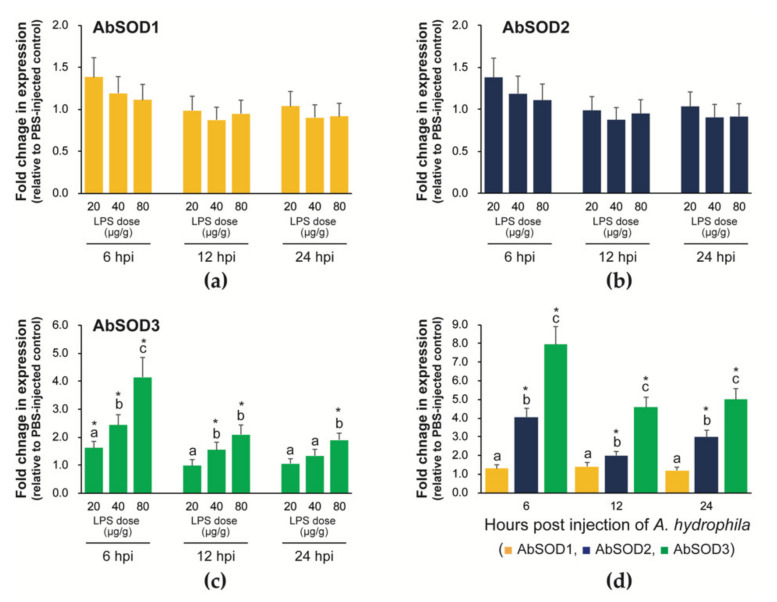
Transcriptional responses of AbSOD isoforms to LPS (**a**–**c**) or *A. hydrophila* (**d**) challenge in 1-day-old *A. baerii* prelarvae. Prelarvae were microinjected with LPS (20, 40, or 80 μg/g BW), *A. hydrophila* (1 × 10^3^ CFU/g BW), or PBS (suspension medium with stimulants), and fold changes in expression at 6, 12, and 24 hpi were examined for each AbSOD isoform based on an RT-qPCR assay. With LPS challenge, AbSOD1 and AbSOD2 did not show any detectable changes in their mRNA expression. In (**c**,**d**), means ± SDs with different letters (a–c) for a given time are significantly different according to ANOVA followed by Duncan’s multiple range test at *p* < 0.05. Asterisks indicate a significant induction of AbSOD mRNAs compared to the expression in PBS-injected control groups, based on a Student’s *t*-test at *p* < 0.05.

Prelarval microinjection with LPS at doses of 20, 40, and 80 μg/g did not induce observable changes in AbSOD1 and AbSOD2 expression at any of the three detection points (6, 12, and 24 hpi) (Figure 7a,b). However, unlike that of those two AbSOD isoforms, AbSOD3 expression was significantly induced by the LPS challenge, and the induction was positively related to the dose (Figure 7c). This dose dependence was most clearly found at 6 hpi, at which the increases in AbSOD3 expression induced by 20, 40, and 80 μg/g LPS were 1.6-, 2.4-, and 4.1-fold, respectively. The patterns were broadly similar at both 12 and 24 hpi, although the dose dependence was not as strong as at 6 hpi.

Based on the results from the LPS challenge, we tested the mRNA expression in response to the microinjection of a known bacterial pathogen, *A. hydrophila*. Similar to LPS injection, the *A. hydrophila* challenge did not significantly modulate AbSOD1 expression. However, the AbSOD2 isoform was significantly upregulated by the *A. hydrophila* challenge at all three detection points, with a maximum fold change of 4-fold at 6 hpi, which was different from the findings for the LPS challenge. Meanwhile, AbSOD3 showed the greatest fold changes, with a maximum increase of 8-fold relative to the PBS-injected control at 6 hpi. The fold changes in AbSOD3 expression at the other two time points were also greater compared to those for AbSOD2 (Figure 7d).

#### 3.5.2. Expression in Fingerlings Injected with *A. hydrophila*

**Figure 8:**
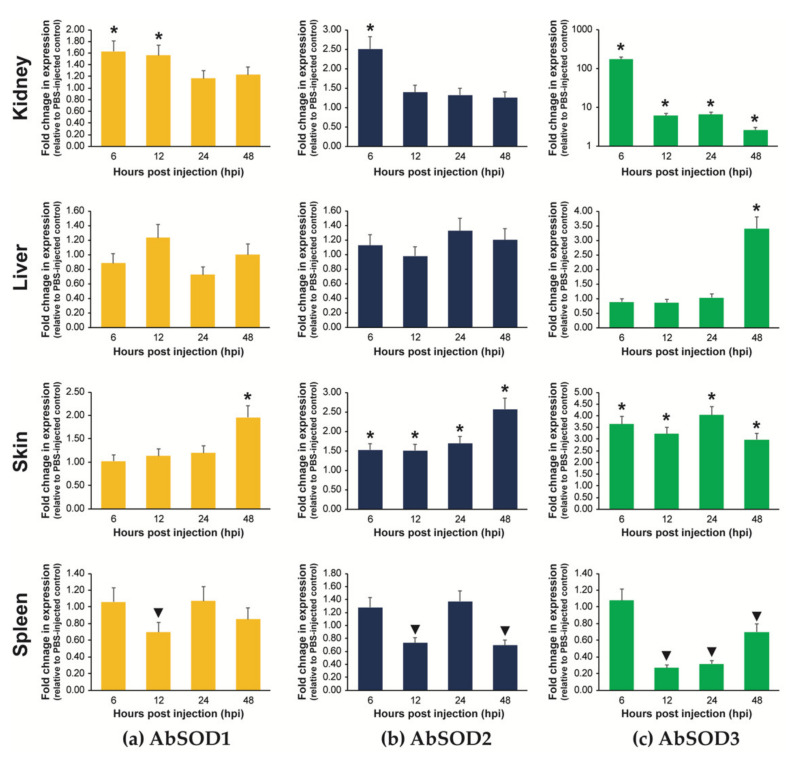
Transcriptional modulation of three AbSOD isoforms (**a**–**c**) in response to *A. hydrophila* challenge (2 × 10^4^ CFU/g body weight) in kidneys, liver, skin, and spleen of *A. baerii* fingerings at 6, 12, 24, and 48 hpi. Asterisks and inverted triangles indicate significantly up- and downregulated expression in *A. hydrophila*-challenged groups, respectively, when compared to expression in PBS-injected control groups according to a Student’s *t*-test at *p* < 0.05.

The transcriptional response of each AbSOD isoform to *A. hydrophila* infection was highly tissue-specific, and the three isoforms exhibited different response patterns within a given tissue type (Figure 8). In the kidneys, AbSOD1 expression was only moderately induced (about 1.6-fold relative to the PBS-injected control) by *A. hydrophila* injection at 6 and 12 hpi and returned to the control level by 24 and 48 hpi. AbSOD2 also showed a 2.5-fold upregulation at 6 hpi; however, the induced expression subsequently diminished. Similarly, the AbSOD3 isoform showed the highest induction of its mRNA expression at 6 hpi. However, the induced fold change in AbSOD3 expression at 6 hpi was strikingly higher (more than 170-fold) than the changes in AbSOD1 and AbSOD2. Although the induced expression of AbSOD3 at 6 hpi weakened over time, it remained significantly higher at 12–48 hpi (2.6-fold to 6.6-fold) than that in the PBS-injected group. In the liver, the expression of both AbSOD1 and AbSOD2 showed no significant change during the *A. hydrophila* challenge. The hepatic expression of AbSOD3 changed little until 24 hpi but was significantly upregulated, 3.4-fold, at 48 hpi. In the skin, all three AbSOD isoforms were remarkably modulated by the *A. hydrophila* challenge in an isoform-dependent manner. AbSOD1 expression in the skin was moderately induced (2.0-fold) only at 48 hpi, while both AbSOD2 and AbSOD3 were upregulated at all four detection points. The upregulation of AbSOD2 expression was slightly higher at 48 hpi (2.6-fold) than earlier times (less than 1.7-fold). The fold changes in AbSOD3 expression in the skin ranged from 3.0 to 4.0, and these were greater than those observed for AbSOD1 and AbSOD2. However, unlike for these two, no clear time-dependent pattern was found for the expression of AbSOD3. Finally, in the spleen, significant downregulations of mRNA expression were observed for AbSOD1 (at 12 hpi), AbSOD2 (at 12 and 48 hpi), and AbSOD3 (at 12, 24, and 48 hpi).

## 4. Discussion

Fish are likely to be exposed to conditions that cause harmful ROS-mediated oxidative stress because they adapt to a wide range of habitats and stressful environments associated with their life strategies [15,22,24]. Consequently, it is of particular interest to comprehensively understand how fish have evolved to modulate their antioxidant defense systems in response to the oxidative stress that is generated through diverse mechanisms including normal cellular metabolism, environmental perturbations, and/or pathogenic infections. In this study, we molecularly characterized and studied the gene expression patterns of three SOD isoforms (Cu/Zn-cytosolic SOD, Mn-mitochondrial SOD, and Cu/Zn-extracellular SOD) regarding their antioxidant defense roles associated with development, ontogeny, and infection in the Siberian sturgeon *A. baerii*, the extant primitive fish that occupy the basal phylogenetic position (Chondrostei) of the ray-finned fish group (Actinopterygii).

The results from sequence comparison and 3D structure analysis demonstrated each AbSOD isoform’s respective characteristics, SOD domain(s), motif(s), and metal ion coordination residues—in particular, Cys residues forming intramolecular disulfide bond(s) for AbSOD1 and AbSOD3, which were conserved in vertebrate SOD orthologous groups. Previous biochemical and structural studies have demonstrated that SOD1 exists as a homodimer, while SOD2 and SOD3 exist as homotetramers in their corresponding cellular locations for in vivo antioxidant activity [63,64,67]. According to the best-characterized human SOD1 structure, the homodimer of SOD1 is formed by the connection of two identical subunits through both hydrophobic interactions—by the residues Val^6^, Val^8^, Ile^18^, ^114^IGR^116^, and ^149^VIGIAQ^154^—and electrostatic interactions, by the residues Glu^130^, Glu^131^, Lys^137^, and Thr^138^ [63,68]. These residues are fully conserved in the AbSOD1 protein sequence, with only an exceptional replacement of the Lys^137^ in the human SOD1 with a Val^137^ residue in AbSOD1. The 3D model superimposed with the tertiary human SOD1 structure also suggests that AbSOD1 can perform an antioxidant function as a homodimer. For SOD2, it is known that a helical hairpin structure at the N-terminus and Mn^2+^ ion coordination residue (His^163^) play a key role in the formation and stabilization of the tetrameric structure [69]. AbSOD2 displays not only a highly homologous sequence at the N-terminus that forms a helical hairpin (only one substitution for Gln^21^ in the human SOD2 was replaced with Glu^21^ in AbSOD2) but also a similarly predicted helical hairpin structure including the conserved His^163^ residue, collectively suggesting that AbSOD2 also functions as a homotetramer in the mitochondrial matrix. A previous study on human SOD3’s tertiary structure demonstrated that a functional homotetramer is fashioned through contacts between two loops (β1, ^41^LHAACQVQPS^50^, and β2, ^75^LDAFFALEGFP^85^) on each subunit [70]. Moreover, although the tertiary structure at both the N- and C-terminal ends of human SOD3 was incompletely resolved, it suggested that human SOD3 requires both the N- and C-terminal ends for tetrameric oligomerization [70]. Notably, it has been found through circular dichroism (CD) spectral analysis, that the N-terminus of SOD3, particularly residues Ala^14^ to Met^32^ in human SOD3, form an amphipathic α-helix that is responsible for tetrameric oligomerization, [71]. Indeed, point mutational analysis of rat dimeric SOD3 compared with other tetrameric SOD3s demonstrated that the substitution of Asp^24^ with a Val^24^ residue (numbering based on human SOD3) converted the form from dimeric to tetrameric [72]. The two loop sequences (^38^LVYASCEMK^46^ and ^73^LESIINLEGFP^83^) in AbSOD3 were comparable to human SOD3 when superimposed on the 3D structure. Strikingly, we found that the Val^18^ residue (^17^KVND^20^) in AbSOD3, corresponding to the Val^24^ residue (^23^KVTE^26^) in human SOD3, appeared to be conserved in the N-terminus, suggesting that AbSOD3 may act in a tetrameric form to eliminate ROS in the extracellular matrix in vivo. However, multiple sequence alignments revealed that most teleostean SOD3s had short N-terminal lengths, which are thought to be insufficient for forming α-helices at the N-termini, and the Val residue was not detected in teleostean orthologs. Therefore, further biochemical studies on whether teleost fish SOD3s form homotetramers for in vivo antioxidant activity are of interest. The SOD family is one of the oldest enzyme classes, whose occurrence dates back to the era of primitive Earth (no later than the Great Oxidation Event, 2.4 GYA), evolving in ancient life as a means of protecting against O_2_ toxicity in a reducing environment [10]. Due to their vastly prolonged existence and widespread presence in most modern-day life forms, the divergence and convergence of SOD isoforms have long received attention in various evolutionary studies. The emergence and distribution of different SOD isoforms throughout all kingdoms of life have been valuably described in several previous studies, and currently, the hypothesis of the occurrence of a very primitive version of SOD in ancient organisms, followed by divergence into modern lineages driven by a wide variability in chemical environments, has been widely accepted [10,12]. However, despite the growing wealth of information on the evolutionary landscape of SODs across life branches, high-resolution phylogenetic analyses of SOD isoforms in fish remain insufficient. Actinopterygians (ray-finned fish belonging to the superclass Actinopterygii) are the largest and most diverse group of vertebrates, making up roughly half of all extant vertebrate species [13]. They exhibit remarkable biodiversity with respect to morphology, habitat adaptation, migratory behavior, and many other physiological aspects. The diversity of actinopterygians (particularly teleosts) has been attributed to whole-genome duplication (WGD) events, a high rate of chromosomal rearrangements, and fast-evolving protein sequences [13,14]. Indeed, the evolution of SOD (Cu/Zn SOD) in certain fish species has been proposed to be closely related to antioxidant defenses associated with habitat adaptation, and this protein represents “unclock-like” and/or “erratic” behavior in molecular evolution in Antarctic species adapted to sub-zero water temperatures [22,73]. From this point of view, the present phylogenetic data are meaningful in that they provide genetic information for potential prototypes of ray-finned fish (Actinopterygii) SOD isoforms, which may be a good starting point for predicting how evolution has accommodated differences in the shapes and functions of SOD isoforms in actinopterygian lineages. In the present phylogenetic analyses, the tree topology of the SOD1 isoform, which is known to be the latest version of SOD, was broadly in agreement with known taxonomic appraisals, including the formation of a monophyletic clade composed of actinopterygian members, in which AbSOD1 formed a subclade together with a holostean ortholog, although the sarcopterygian group was not resolved as a monophyletic clade (i.e., the separation between lobe-finned fish and tetrapods). SOD2 (Mn-SOD) is the oldest isoform in vertebrates and is thought to have originated very early in primitive lifeforms and share a common ancestor with Fe-SOD, the most ancient SOD form [10]. The branching topologies of the SOD2 phylogenetic tree presented in this study are fairly congruent with the known taxonomic evaluations. They include the formation of monophyletic sister clades of actinopterygian and sarcopterygian orthologs, the outmost position of chondrostean SOD2 (sturgeon, i.e., AbSOD2) in the actinopterygian clade, and the holostean–teleostean relationship resolved in the subclass Neopterygii. Furthermore, within the infraclass Teleostei, the branching patterns of subclades are also generally in agreement with those previously suggested for the phylogenetic classification and the speciation of bony fish, suggesting that SOD2 could be a candidate phylogenetic marker for addressing the evolutionary history of actinopterygians [74,75]. However, unlike the SOD1 and SOD2 isoforms, the evolutionary path of the SOD3 isoform hypothesized from the present phylogenetic trees is somewhat questionable. Although teleostean SOD3s formed a monophyletic clade with bootstrap support, not all the actinopterygian orthologs were monophyletically recovered; the SOD3s from basal actinopterygians (non-teleosts such as gar, sturgeon, and reedfish) were more closely affiliated with those from sarcopterygians, including coelacanth and non-mammalian tetrapods (amphibians, reptiles, and birds), than with orthologs from relatively recently evolved actinopterygians (teleosts). From an evolutionary standpoint, our findings suggest that the evolution and diversification of SOD3s might be discordant with the generally established phylogenetic framework for early osteichthyans, which is essentially based on the actinopterygian–sarcopterygian split during the early Devonian period (about 419 MYA) [76]. SOD3 (extracellular Cu/Zn-SOD) is considered to be a more ancient isoform than the intracellular version (SOD1) based on the phylogenetic result that mammalian SOD3s are more closely related to fungal Cu/Zn-SODs than to other animals’ SOD1s [77]. However, the evolutionary history of the SOD3 isoform in the vertebrate lineage is not yet comprehensively understood. Thereby, further extensive studies are needed to gain deeper insights into the evolutionary repertoire of SOD3 compared to that of the SOD1 and SOD2 isoforms in the actinopterygian lineage.

Although it is known that the relation between SOD expression and its enzymatic activity was not strictly linear, the expression of SOD1 and SOD2 mRNAs in various tissues have been reported to be generally correlated with their activities in various fish species [27,78,79,80,81]. The ubiquitous detection of all three AbSOD isoform transcripts across diverse tissue types, based on the tissue expression analysis, was unsurprising and in good agreement with their housekeeping roles in scavenging radicals [8]. However, the tissue distribution patterns of the basal expression levels of the three different AbSOD isoforms were rather variable according to tissue type. Unfortunately, there are few studies to directly compare with our results, but one study found that the distribution patterns for all three SOD isoforms in the yellow croaker *Pseudosciaena crocea* seemed to be similar across tissue types; all the isoforms displayed the highest expression in the liver, followed by the kidneys, spleen/gills/brain, and intestines [23]. Nevertheless, there are several studies of only one or two fish SODs, excluding SOD3, in terms of tissue distribution patterns. For example, the tissues in descending order for SOD1 expression were the spleen, gills/muscles, heart, and liver in the crocodile icefish *Chionodraco hamatus* and the kidneys, brain, liver/heart/gills, and stomach in the yellow drum *Nibea albiflora* [22,24]. The tissues in descending order for SOD2 expression were the gills, spleen, liver, and heart in the silver carp *Hypophthalmichthys molitrix* and the liver, gills, muscles, and spleen in the blunt snout bream *Megalobrama amblycephala* [25,26]. In the fingerling stage of *A. baerii*, the liver was the main site of expression for the AbSOD1 and AbSOD3 isoforms, and similar results were also found in previous studies on other teleosts including the marbled eel *Anguilla marmorata* SOD1 and the *P. crocea* SOD1 and SOD3 [23,27]. Based on the general consideration that the fish liver is the primary organ responsible for detoxification and stress responses [82,83], it demands significant antioxidant activity under normal growing conditions during the fingerling stages of *Acipenser* species. Conversely, AbSOD2 expression was the highest in the heart but only moderate in the liver, which is similar to the pattern observed in a cypriniform species, *Hemibarbus mylodon* [28]. Considering the fact that the major tissue of SOD3 expression in human is the lung [20], it is surprising that AbSOD3 was rarely detected in the gill, the central respiratory system of fish. Although there is no clear experimental evidence yet, the difference in the expression levels of SOD3 in the two organs (human lungs and fish gills) is probably related to the adaptation to different oxygen concentrations between the air and aquatic media. Possibly, terrestrial animals have evolved the specific antioxidant system with SOD3 to protect their respiratory organ against the oxygen toxicity caused by the high concentration of oxygen in the terrestrial atmosphere. Collectively, these results suggest that each organ (or tissue) in fish has a different metabolic profile and variable energetic demands due to inherently different functions [84]. The tissue distribution patterns of the basal expression of the three different fish SOD isoforms could be species-specific for antioxidant defense under normal physiological conditions.

Furthermore, we sought to quantify and compare the relative proportions of the three AbSOD isoforms in each tissue to understand the complexity of their expression patterns, though we did not estimate the absolute copy numbers of each AbSOD RNA species. AbSOD1 was the predominant isoform in most tissue types, which is consistent with a previous study reporting that SOD1 (Cu/Zn-SOD) is a major and bulk scavenger of radicals [8]. Each animal organ or tissue maintains a specific capacity for oxidative phosphorylation (OXPHOS—the process of ATP production in mitochondria relying on aerobic metabolism) to satisfy its metabolic roles (the contribution of OXPHOS to the main metabolic pathways) [85], implying potential differences between tissues in the mitochondria, such as number, function, and protein composition [84]. Recently, it has also been reported that the tissue-specific features of mtDNA maintenance are primarily driven by intrinsic ROS exposure in mammals [86]. Within this context, the relatively high proportion of AbSOD2 abundance shown in a variety of tissues, including the heart, brain, and muscles, which are known to have high OXPHOS activity, is potentially indicative of the important roles of mitochondrial SOD (SOD2) in the protection of the mitochondrial matrix against oxidative stress [86,87]. However, AbSOD3 appeared in only the liver, with a relatively high proportion of abundance, which is probably associated with the fact that SOD3 remains localized to tissues for its expression and secretion and is coregulated along with factors that are important in tissue localization [88].

Sturgeon, like most other fish species, exhibit oviparity in their embryonic development, implying that fertilized embryos develop their antioxidant defense systems independently of parental contribution during their early development. Several previous studies have proposed important roles for SOD in the development of fish embryos, including different teleost species—*Salmo iridiaeus* [29], *Oncorhynchus mykiss* [30], *Scophthalmus maximus* [89], *Lates calcarifer* [90], *Oryzias latipes* [91], and *Austrofundulus limnaeus* [32]—as well as sturgeon species: *A. gueldenstaedtii* and *A. naccarii* [31,44]. These studies suggest that the AOE system is finely regulated in response to changes in the oxidative stress status during early development, including from cell proliferation and specific signaling for organogenesis, although the specific temporal patterns are little generalized due to species-specific differences. Such differences might be related to many intrinsic factors including the composition of the eggs, developmental speed, habitat environment, and capacities to tolerate temperature changes, anoxia, or pollutants [19]. Sturgeon eggs, particularly in *Acipenser* species, have been shown to contain substantially high proportions of polyunsaturated fatty acids in the lipid fractions of their yolks [92,93], suggesting that antioxidant defense systems are important for protecting the developing embryos against oxidative stress driven by lipid peroxidation.

However, most of the previous studies mentioned above have come to conclusions based on enzymatic measurements of total or pooled activities of SODs, without distinguishing between the three SOD isoforms, making it difficult to hypothesize the roles of specific SOD isoforms in certain developmental periods. Based on our findings, the main isoform responsible for antioxidant defense throughout embryonic development in *A. baerii* is AbSOD1. This isoform is thought to actively respond to increasing antioxidant demand, particularly in the phase of robust cell proliferation (i.e., uneven holoblastic cleavages followed by blastocoel formation [40]) and in the phase for the preparation of hatching out. On the other hand, the role of AbSOD2, the second major isoform, in development seems to be more important in the late developmental stage close to hatching than the initial cleavage stage. Meanwhile, AbSOD3 showed a specific spike only within a narrow window of the stage, which corresponds to the period for the preparation of the heart and blood vessels in this sturgeon species [40,42]. During this period, *A. baerii* embryos show a differentiation of a functional heart (called the “S-heart”) and blood vessels in preparation for the onset of blood circulation [40]. Considering the many mammalian studies that highlight the crucial roles of SOD3 in vascular protection [94,95,96], the spike unique to AbSOD3 at this stage might reflect strategic upregulation in preparation for SOD activity in relation to the vascularization process. Although we did not measure SOD enzyme activity, our data on the temporal changes in mRNA expression are broadly in agreement with the results for enzyme activity from a closely related species, *A. gueldenstaedtii* [44]. However, our findings were largely in contrast to a previous observation of *A. naccarii* embryos, where the pooled SOD activity was rapidly decreased with development [31].

After hatching out, *A. baerii* hatchlings spend a relatively long period (up to 10 days at 18–20 °C) of prelarval development undergoing dynamic changes in not only their morphology (metamorphosis and organ differentiation) but also behavioral patterns [60]. According to our expression data, AbSOD1 might have a continuing role as the predominantly expressed isoform in antioxidant defense during the prelarval period of this sturgeon species, and AbSOD2 exhibits a temporal expression pattern that broadly resembles that of AbSOD1. Overall, the modulation pattern of AbSOD1 (and possibly AbSOD2) appears to be closely related to behavioral modifications in *A. baerii* prelarvae. During the early ontogenetic period (1 to 3 dph), showing an initial increase in the expression of AbSOD1 (and AbSOD2), *A. baerii* prelarvae change their behavior from drifting movement to active pelagic swimming at the upper water column, with dramatic increases in swimming distance and speed. Furthermore, prelarvae during this period have the strongest positive phototaxis, making them continuously pursue light sources [61]. Undoubtedly, such energy-intensive behavioral changes are accompanied by an increased respiration rate and parallel accumulation of oxygen radicals. After 3 dph, *A. baerii* prelarvae begin to show another notable change in their behavior: a transition from pelagic swimmers to bottom swimmers. During this period (usually at 5–6 dph), very strong rheotaxis is also acquired in prelarvae, which is characterized by a continuous and vigorous propelling movement against water currents [60]. Thus, the peak in AbSOD1 expression at around 5 dph could be interpreted as strategic upregulation to address the oxidative stress caused by such energy-intensive exercise. Thereafter, prelarvae exhibit a typical “schooling” behavior at the corners of a tank, with a progressive weakening of rheotaxis (7 dph), followed by “post-schooling rest” at 9 dph characterized by non-locomotory, scattered, and sedentary settlement at the bottom [60]. In accordance with behavioral modifications, the gradual decreases in expression from either 5 (for AbSOD1) or 7 dph (for AbSOD2) could reflect the decreased demand for antioxidant responses owing to the reduced activity and energy-saving behavior. After the evacuation of the pigment plug at 9–10 dph [39,97], prelarvae enter the larval stage, with a transition from endogenous nutrition to exogenous feeding (11 dph), and their locomotor activity begins to recover, which is in agreement with the rebound in SOD expression (for both AbSOD1 and AbSOD2 at 11 dph) to prepare the antioxidant defenses for increased metabolic rates.

However, unlike that of AbSOD1 and AbSOD2, the temporal expression pattern of AbSOD3 exhibits a weaker interrelationship with the behavioral modification pattern; the slope of its elevation in the early ontogenic period is much gentler than the slopes observed for the other two isoforms. Furthermore, after reaching a peak at 7 dph, the mRNA levels tend to be fairly stable until 11 dph, although there was a slight decrease at 9 dph. We have not yet clearly hypothesized the mechanism behind the pattern of AbSOD3 expression. However, one plausible explanation is that the continuous increase in this isoform might be partially related to neovascularization and increased circulation activities in developed blood vessels, rather than to the defense against the overall oxidative stress caused by high-oxygen-consuming exercise. Previous studies have indicated that *A. baerii* hatchlings usually continue vasculogenesis/angiogenesis during the prelarval period, especially associated with the active development of the external gill filaments and branchial circulation [39,40,42,61].

In the present study, the microinjection-mediated delivery of an immunostimulant or pathogen was successful in inducing oxidative stress in sturgeon prelarvae. Larval microinjection has already been considered to be a useful tool for studying bacterial pathogenesis, virulence, and immune responses, although most of the relevant studies have used the zebrafish model [98,99,100]. The transcriptional regulation of the AbSOD isoforms in the microinjected prelarvae in response to LPS and/or *A. hydrophila* challenge appeared to be different depending on the isoform type, and overall, the most sensitively upregulated isoform was AbSOD3. Considering the temporal expression pattern after immune stimulation, SOD-mediated antioxidant defenses could be quickly activated in *A. baerii* prelarvae as an acute response (as early as 6 hpi) to microbial invaders. However, a further study should be conducted to analyze the main site(s) of expression in the prelarvae responsible for the prompt immune/antioxidant response, since the microinjected prelarvae do not exhibit complete organogenesis of immune-relevant organs at this age [39,42]. Although the larval expression patterns of the three different AbSOD isoforms during experimental immune challenge have been seldom studied, the acute and early response patterns of the AbSODs observed in this study are congruent with previous observations made in LPS- and *A. hydrophila*-challenged pufferfish, *Takifugu obscurus* [33].

In addition to the prelarval experiments, the *A. hydrophila* challenge with the fingerlings highlighted not only the isoform-dependent modulation of AbSOD but also the tissue dependency of AbSOD regulation during pathogen infection. The fish kidney is the main erythropoietic tissue and known as one of the key immune organs [101]. During the *A. hydrophila* challenge, all the three AbSOD isoforms exhibited acute and early response patterns (at 6 hpi) in the kidneys. However, the extent of induction greatly differed among the AbSOD isoforms; the minute or moderate induction of AbSOD1/AbSOD2 in contrast to the very robust upregulation of AbSOD3 suggests that the sensitive regulation of AbSOD3 is important for early protection against infection-mediated oxidative stress in the kidneys. The bacterial pathogen *A. hydrophila* is known to cause serious hemorrhagic septicemia in many species, including sturgeon, and the kidneys are one of the main targets [46,47,102]; the rapid induction of AbSOD3, a major isoform for vascular protection [96], could be tactical preparation by the host in response to the damage to blood vessels and blood lysis. Because hemorrhages often release large amounts of free heme, which is known to be a strong pro-oxidant and pro-inflammatory molecule [103,104], the involvement of SODs in protecting against hemorrhage-associated oxidative stress might also be important in this erythropoietic organ.

The fish skin is the primary line of defense against invading pathogens in the mucosal innate immune system, constituting various inherent components involved in both humoral and cellular immunity, including antimicrobial responses and cytokine production [105,106]. Such immune responses to invading pathogens require a balanced mechanism of antioxidant defense to protect the tissue from oxidative damage. Therefore, our findings of the robust induction of SOD expression in the skin of *A. hydrophila*-infected sturgeon is generally in agreement with the proposed crosstalk between the immune response and antioxidant defense [8,107]. From a pathological perspective, a typical sign of *A. hydrophila-*induced septicemia in fish is hemorrhaging on the skin surface [47,102,108], suggesting a requirement for SOD function in the skin similar to that in the kidneys. However, the temporal patterns of SOD expression in the skin (AbSOD1 and AbSOD2) were relatively delayed compared to the acute response observed in the kidneys, which might be due to the order in which the tissues are affected with the progression of hemorrhagic pathogenesis. Conversely, AbSOD3 displayed higher fold changes than the other two isoforms throughout the examination period, again suggesting that AbSOD3 is the most sensitively modulated isoform in the skin. Collectively, the results from this study are congruent with the induced expression of SOD in the skin or isolated epidermal cells during bacterial infection in the Atlantic cod *Gadus morhua* [109,110], although the isoform dependency of SOD regulation was not examined in those previous studies.

Unlike in the kidneys and skin, the transcriptional modulation of the AbSOD isoforms during *A. hydrophila* infection were not robust in the liver. A significant induction was only observable for AbSOD3 at a single time point (48 hpi). The late response of hepatic AbSOD3 expression during bacterial challenge is similar to findings from the liver of *Vibrio alginolyticus*-challenged yellow croaker *P. crocea*; however, significant induction of the hepatic expression of the two other SOD isoforms upon the same challenge have been reported [23]. Our findings are also in contrast to previous observations made with *A. hydrophila*-challenged *A. marmorata* and *A. hydrophila*-challenged *T. obscurus*, which showed significant and early responses of the SOD1 and SOD2 isoforms to the bacterial pathogen in the liver [27,33]. The isoform specificity and tissue dependency of SOD expression during bacterial infection may also be largely species-specific.

The spleen is also considered a main immune organ in teleostean fish species [111], and the chondrostean sturgeon has also been reported to exhibit active innate immune/inflammatory responses in the spleen during bacterial invasion [36,37]. However, in the present study, induced expression in the spleen was observed for none of the AbSOD isoforms during *A. hydrophila* challenge—instead, it was suppressed. The splenic expression of SODs in response to bacterial challenge appears to be inconsistent and contradictory depending on the fish species. The lack of splenic upregulation in this study is broadly similar to findings from *A. hydrophila*-challenged yellow catfish, *Pelteobagrus fulvidraco* [112], and with the suppressed expression of immune/stress-responsive genes in the carp *Cyprinus carpio* during *A. hydrophila* challenge [113]. However, our findings are in contrast to other previous results from tilapia, *Oreochromis niloticus* [114]; yellow drum, *N. albiflora* [115]; and Korean spotted barbel, *H. mylodon* [116], which all suggest that the splenic upregulation of SOD is involved in antioxidant defense during bacterial infection. Because we were able to observe the significant induction of several marker genes involved in inflammation, antimicrobial defense, and/or iron sequestration (such as interleukin-1β, hepcidin, and transferrin) in the spleens of challenged individuals (data not shown), it may be assumed that the combating of infection-driven oxidative stress might not rely so much on the induction of AbSOD transcription in the spleens of *A. baerii* fingerlings. Alternatively, based on the observation that *A. baerii* fingerlings have the lowest basal levels of all three AbSOD isoforms, the acute damage caused by *A. hydrophila* might be beyond the capacity of splenic regulation to promptly prepare the upregulation of AbSODs, which may be related to our findings on the suppression of SOD isoforms in the spleen during *A. hydrophila* challenge. Further studies examining the effects of biotic and abiotic factors on the capacity for splenic SOD regulation in this sturgeon species could be conducted, particularly regarding differential responses according to different grow-out stages, since the capacity and sensitivity of the antioxidant enzyme systems in fish organs can vary considerably with age [19,31,44].

## 5. Conclusions

Three SOD isoforms (AbSOD1, AbSOD2, and AbSOD3) were characterized from a chondrostean sturgeon species, *A. baerii*, and their transcriptional regulation patterns were comparatively investigated regarding embryonic development, prelarval ontogeny, and immune responses. Each isoform of *A. baerii* SOD shared conserved features in its deduced protein structure and key motifs with its vertebrate orthologs. Molecular phylogenetic analyses indicated that each *A. baerii* SOD isoform potentially represents a basal prototype of actinopterygian SODs, but also that the AbSOD3 isoform has an evolutionary history distinct from that of SOD1 and SOD2. The tissues showing the predominant SOD expression were the liver (AbSOD1 and AbSOD3) and heart (AbSOD2) in *A. baerii*. During embryonic development, the AbSOD isoforms were dynamically modulated in an isoform-dependent manner, but AbSOD1 was always dominant in quantitative terms. The regulation of the AbSOD isoforms during the prelarval period was closely related to the behavioral modifications or transitions of the prelarvae, mainly according to oxygen-/energy-demanding activities and exercise. Prelarval microinjection experiments showed that *A. baerii* prelarvae were able to promptly induce SOD transcription in response to exogenously delivered LPS and *A. hydrophila*. Upon the *A. hydrophila* challenge in fingerlings, the SOD isoforms were significantly regulated in a tissue- and isoform-dependent fashion. Overall, the isoform most sensitively regulated during the immune challenge was AbSOD3, and its upregulation was the greatest in the kidneys and skin. Taken together, the results from this study reveal the isoform-dependent regulation of multigene SOD members in the preparation of antioxidant defenses against the oxidative stress associated with development and immune responses in *A. baerii*, which could be a useful basis for better understanding the ability of this primitive actinopterygian to maintain a homeostatic pro-oxidant and antioxidant balance during its early life.

## Data Availability

Data is contained within the article.

## Supplementary Materials

The following are available online at https://www.mdpi.com/2076-3921/10/2/232/s1. Figures S1–S9; Tables S1 and S2.
