# Supplementary material for: Superoxide Dismutase Multigene Family from a Primitive Chondrostean Sturgeon, Acipenser baerii: Molecular Characterization, Evolution, and Antioxidant Defense during Development and Pathogen Infection
Source: Antioxidants (Basel). 2021 Feb 3;10(2):232. doi: 10.3390/antiox10020232 (PMC7913737; doi:10.3390/antiox10020232)
Supplement: Supplementary file 1 [file antioxidants-10-00232-s001.zip › Supplementary Figures.docx]

1

48

123

198

273

348

423

498

573

648

723

798

873

948

attcaaaaccctgacgactggctgtgcggagctgaagattgaacttg

ctggtttagcctgtacagtttttgcagagatttttgaacggtgctggatttacactgaaactgcagtcagaaaat

ATGGTGTTGAAAGCTGTTTGCGTTCTGAAGGGCACCGGCGACGTCTGTGGAACGGTGCATTTTGTGCAAGAAAAG

**MVLKAVCVLKGTGDVCGTVHFVQEK**

GAGGCTGGACCAGTGAAGTTAACGGGGCAAATAACAGGTTTAACTCCTGGAGAGCATGGCTTTCACGTCCATGCA

**EAGPVKLTGQITGLTPGEHGFHVHA**

TTTGGAGACAACACCAATGGTTGTGTGAGTGCTGGTCCTCACTTCAACCCACTTGGCAAAACCCATGGTGCGCCG

**FGDNTNGCVSAGPHFNPLGKTHGAP**

CAAGATGAAATTAGGCATATAGGAGATCTTGGTAATGTAATAGCTGGAGATGATAAGGTGGCAATTATTAATATC

**QDEIRHIGDLGNVIAGDDKVAIINI**

GAGGACAAACTTATAACTCTATCAGGAGCTTATTCAATCATAGGTCGAACTATGGTGATCCACGAGAAAGCTGAT

**EDKLITLSGAYSIIGRTMVIHEKAD**

GATTTGGGCAAAGGAGGAAATGACGAGAGCCTGGTGACTGGCAATGCTGGTGGCCGCTTGGCCTGCGGAGTAATT

**R**

**DLGKGGNDESLVTGNAGGRLACGVI**

GGAATTGCTCAAAGCTAAtcttcaatgtttctgtcttcattccaaatagtggcacttatactagaatttagtaga

**GIAQS***

tattgtagggcaatatcaaagcccagtacaaagtaaagcacttgctgattagtgtggttttctttaatgaaaaaa

taattgttttgcgtgttagaaatcactaataataaaccttaaaatagctgtgtgccgtgtaagtgccatctgaaa

atgtcacagatttatattagtatattttgttcaaggtaatggccattatgccctgaaatgttgcagcatttacat

aacattgaatgtatgttcgatatatattttgctgtatggaataaccaaataaactggtgcaacttaaaaaaaaaa

aa

25

50

75

100

125

150

155

**Figure S1**. Full-length nucleotide cDNA sequence and its deduced amino acid sequence of AbSOD1. The protein sequence (uppercase, 155 amino acid residues) is shown below the cDNA coding sequence (lowercase, 9493 bp). A polyadenylation consensus sequence is double underline in the cDNA sequence and the stop codon (TAA) is indicated with an asterisk (*). The Cu/Zn SOD domain is shown in green letters, where the Cu/Zn-binding site is underlined. Two Cu/Zn-SOD motif signatures are shown in the yellow highlighted amino acid residues. Four blue squares and four red circles indicate the residues for Cu^2+^ ion and Zn^2+^ ion coordination, respectively. The two cysteine residues likely form an intramolecular disulfide bond (Cys^58^–Cys^147^) are shown in highlight purple. The residue (Arg^144^) considered as an importance for enzyme activity is shown in red highlighted white letter.

1

44

119

194

269

344

419

494

569

644

719

794

869

944

1019

aagggcagttttcgctgtgtaaatgagagtctgacactgcgac

agactcagagacaggattttcaagttatctcatacttaaatatatgtgttttgttttaacaaaataagttcagat

ATGTTGTGCAGAGTTGGTAGTGTGGGCAGATGTGCCACCAATTTGAGTCCAGCTCTAAGCTGTGTTGCTTCAAGA

**MLCRVGSVGRCATNLSPALSCVASR**

CAGAAGCACACTCTGCCTGACCTGCCTTATGATTATGGTGCACTGGAGCCCCACATTAGCGCAGAGATTATGCAA

**QKHTLPDLPYDYGALEPHISAEIMQ**

CTGCATCACAGTAAGCATCATGCAACATACGTTAACAATCTGAATGTTGCAGAAGAGAAATATCAAGAGGCACTA

**LHHSKHHATYVNNLNVAEEKYQEAL**

GCCAAGGGAGACGTTACAGCACAGATCGCTTTGCAGCCTGCTTTGAAATTTAATGGTGGTGGTAACATCAATCAC

**AKGDVTAQIALQPALKFNGGGNINH**

TCTATTTTCTGGACTAACCTTTCCCCCAATGGTGGTGGAGAACCACAAGGGAAGCTAATGGAAGCTATCAAGCGT

**SIFWTNLSPNGGGEPQGKLMEAIKR**

GATTTTGGCTCCTTTGAGAAGATGAAGGAGAAGCTAGCGGCAGTCTCTGTTGGTGTCCAGGGATCAGGTTGGGGC

**DFGSFEKMKEKLAAVSVGVQGSGWG**

TGGCTGGGGTTCAACAAGGAAAGTGGACGACTAAACATTATTGCCTGCCCTAATCAGGACCCCCTACAAGGAACA

**WLGFNKESGRLNIIACPNQDPLQGT**

ACAGGTCTCACCCCTCTGCTTGGAATTGATGTTTGGGAGCATGCCTATTACCTGCAGTATAAAAATGTGAGGCCT

**TGLTPLLGIDVWEHAYYLQYKNVRP**

GATTATGTAAAAGCGATCTGGAACGTGGTCAACTGGGAAAATGTAGCTGAAAGATTCCAGGCTGCAAAGAAGTAA

**DYVKAIWNVVNWENVAERFQAAKK***

atgagtccaaatgtccattactatgttgctttaaaaaaaaaaaaaaaaaaaaagcacaactgcggctgtgaacat

tttgttagcactcagttcaaatgcacaaatcattacttgtttgcccagctaaagaagtggttaactagcttcaat

gtatgctcacgactctgttttgaaatgttgatagtctatattatatgatgttaatgtttcaataaaagtaatttc

agcaaaaaaaaaaaa

25

50

75

100

125

150

175

200

224

**Figure S2**. Full-length nucleotide cDNA sequence and its deduced amino acid sequence of AbSOD2. The protein sequence (uppercase, 224 amino acid residues) is shown below the cDNA coding sequence (lowercase, 1033 bp). A polyadenylation consensus sequence is double underline in the cDNA sequence and the stop codon (TAA) was indicated with an asterisk (*). The predicted mitochondrial transfer peptide (MTP) is shown in bright blue letters. The Mn/Fe-SOD N-terminal domain and Mn/Fe-SOD C-terminal domain flanked by a central space are shown in green letters, in which the Mn/Fe SOD-biding site is underlined that is identical with the highlighted Mn-SOD signature. The potential *N*-glycosylation site is shown in red letter with a red triangle and double underline. Four blue squares indicate the residues for Mn^2+^ ion coordination.

1

15

90

165

240

315

390

465

540

615

690

465

840

915

990

1065

1140

1215

1290

1365

1440

1515

1590

1665

1740

1815

1890

agctgtccgggctg

aggagctgatgaggctgcagcgtattgactgtgaattgaaaaacagtacttctgtaaaggagtagaaaaacaaca

ATGACGATGTCAGCATTCAGTTTTCTCCTTGCTCTGGCCATCGCTGGAACCCACGTGTCACACAGTGAAGAATCT

**MTMSAFSFLLALAIAGTHVSHSEES**

CCAACATCTGAGGAGAACACAATGAAAAACATAGAGTCCAAAGTGAACGATTTATGGCAGAGCCTGCTTCATCCA

**PTSEENTMKNIESKVNDLWQSLLHP**

GTGGCCTTCGTAGCAAAAGATGCAGAATTAGTGTATGCATCATGTGAAATGAAACCAAGCACCAAACTGGAAGAA

**VAFVAKDAELVYASCEMKPSTKLEE**

GGCAAACCACAAGTTACCGGGAAGGTGCTTTTCAAGCAAGCTTACCCTCAGGGGAGACTGGAAAGCATCATTAAC

**GKPQVTGKVLFKQAYPQGRLESIIN**

CTGGAGGGATTCCCCAAAACAAGTAATCAGTCAAGAGCGATCCACATTCATGAGTTTGGGGACCTCAGCGATGGC

**LEGFPKTSNQSRAIHIHEFGDLSDG**

TGTGATGCTGCCGGAGGTCATTTCAATCCATTCAAGGTTAATCATCCAAGGCATCCAGGTGATTTTGGCAACTTC

**CDAAGGHFNPFKVNHPRHPGDFGNF**

TTGCCCAAAAACAGCCAGATAAAAACATTAAAGAAAAACATCCAAGCAACTATGTTTGGACCTAATTCGTTCCTT

**LPKNSQIKTLKKNIQATMFGPNSFL**

AGCAGGTCCGTGGTGATTCATGAGCTGAAGGATGATCTTGGGAAAGGAGACAACCCAGCCAGTCTACTGAATGGC

**SRSVVIHELKDDLGKGDNPASLLNG**

AATGCCGGCAAGAGGTTAGCTTGCTGTGTCATCGGAATCAGCAATAAAAACCTCTGGGAGAAAACTTCTCAATCC

**NAGKRLACCVIGISNKNLWEKTSQS**

TTAACCAGCAGCAAGAAGAAGCGAAATGCCAGAGGGCTCGCAAACAAACAAGCCTAGatgtttaggaaccggctt

**LTSSKKKRNARGLANKQA***

atcatttgtctacgtaattaaatgtttggctccagtgcaacgttttcaccatctgtttcagtaaggaatatacaa

tcctgttaatgtcacacaggacaaaggaatacattatacactcatagatatctggcatctgtaataaagtggcta

gcatattttacgttttgggactgtctttcagcaagcctgaactctgggctgcctgattacaaccaccctcctcag

gctgctcatagactgtcacaaagctaaactgcttgtgactttattaaacaggtcaaatatccatgtgtgcagaag

cacaaagtaacatatgttatgggataataaactgacataaaatgtattgttttgcagtggagcacggtctatgtt

ttttttcacatgctttcaatcttttgtattttgtactttaccattacaaaactaatgcagtcatgtttcttacat

tttattaaatgtctgtaacatgtaagacaactattgtggcagatcttctgtgttttgtacaaaaacaacaatcta

gaatcagaaaatccccagccaattaaaaagctccattaaattgtagagccaatcagtgacagaagggcagggaga

tatagttgtaatttgacattcccatccacagtacagattgaatgcaacaatattaaagcagggcataatttaaaa

gtgttaaaatctgttcagaaataaatgcagagatggtgaatggaggccgtctgttcaaggcacattcagttttac

attaaggactttctgaattactaaactacccgtacatacaaaatattttaatgggaaacttaatgaaacagggtt

gtatcgtaaatagcagatgtattttgtctttttaaagctggtcaattcaatcatttctcattctttaccactgtt

aattctaatcatgtgatccagtctattccaaagtttaactgtggatttgttttgaactctatgcaacaggagttg

cagtagtgttactataacagcatattattgcttatactttggtaaatcaaatgcttttgtctacattgttaaaaa

aataaagcatgttttacactttaaaaaaaaaaaa

25

50

75

100

125

150

175

200

225

243

**Figure S3**. Full-length nucleotide cDNA sequence and its deduced amino acid sequence of AbSOD3. The protein sequence (uppercase, 243 amino acid residues) is shown below the cDNA coding sequence (lowercase, 1923 bp). A polyadenylation consensus sequence is double underline in the cDNA sequence and the stop codon (TAG) is indicated with an asterisk (*). The predicted signal peptide (SP) is shown in the bright blue letters. The Cu/Zn SOD domain is shown in green letters, in which the underlined letters indicate the Cu/Zn-binding site. The predicted *N*-glycosylation site is shown in red letter with the red triangle and double underline. The yellow highlighted amino acid residues represent two Cu/Zn-SOD signatures. Four blue squares and four red circles indicate the residues for Cu^2+^ ion and Zn^2+^ ion coordination. The four cysteine residues likely form two intramolecular disulfide bonds (Cys^65^–Cys^209^ and Cys^126^–Cys^208^) are shown in highlight purple.


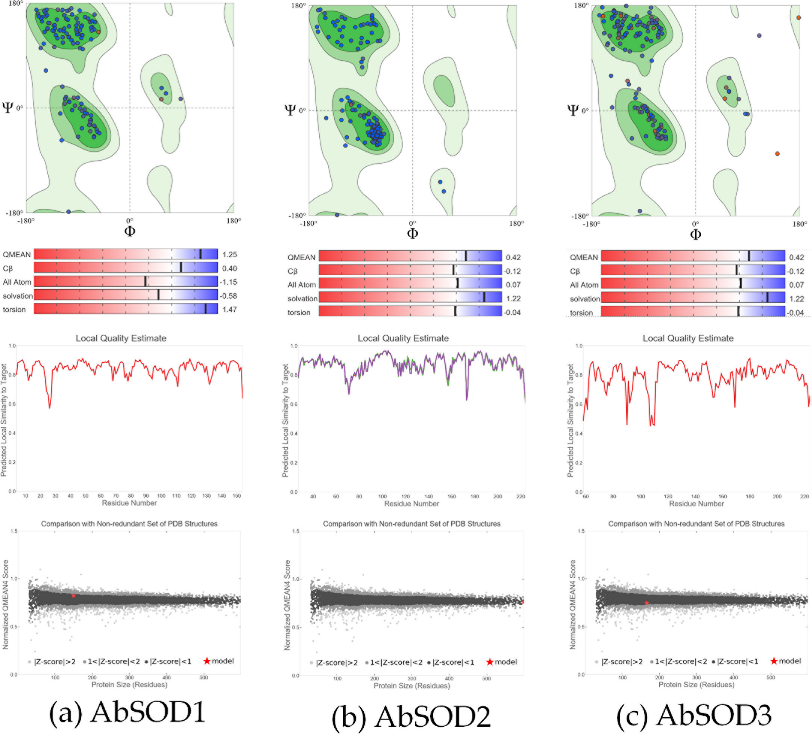


**Figure S4**. Quality estimation of the obtained structural models of AbSOD proteins. Each illustration for each AbSOD model represents Ramachandran plot, QMEAN, local quality estimate, and comparison plot in order from top to bottom. In the Ramanchandran plots, (a) the AbSOD1 protein model showed 1.79% MolProbity score, 98.66% residues were in the favored residues, and 0% in the outliers regions, and the Clash score was 19.46%; (b) the AbSOD2 protein model showed 2.24% MolProbity score, 96.94% residues were in the favored residues, and 0% in the outliers regions, and the Clash score was 15.27%; (c) the AbSOD3 protein model was the MolProbity score of 2.66%, the favored residues of 92.73%, the outliers regions of 1.82%, and the Clash score of 53.12%. The QMEAN Z-scores were 1.25 of the AbSOD1, 0.42 of the AbSOD2, and 0.42 of the AbSOD3. These results indicated that correct folds have been predicted for all AbSOD models.


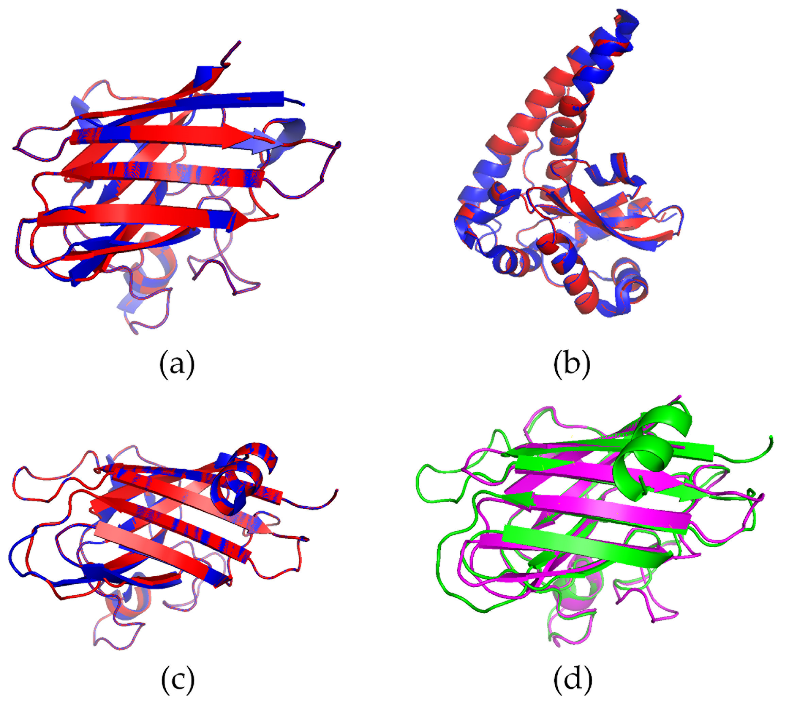


**Figure S5**. Comparisons of the AbSOD models (red colored) with the corresponding human X-ray crystallographic structures (blue colored): (a) the AbSOD1 model was superimposed with human SOD1 (PDB ID: 2C9V); (b) the AbSOD2 was compared with human SOD2 (PDB ID: 1N0N); (c) the AbSOD3 was overlapped with human SOD3 (PDB ID: 2JLP); (d) the AbSOD1 model (magenta colored) was superimposed with the AbSOD3 model (yellow green colored).


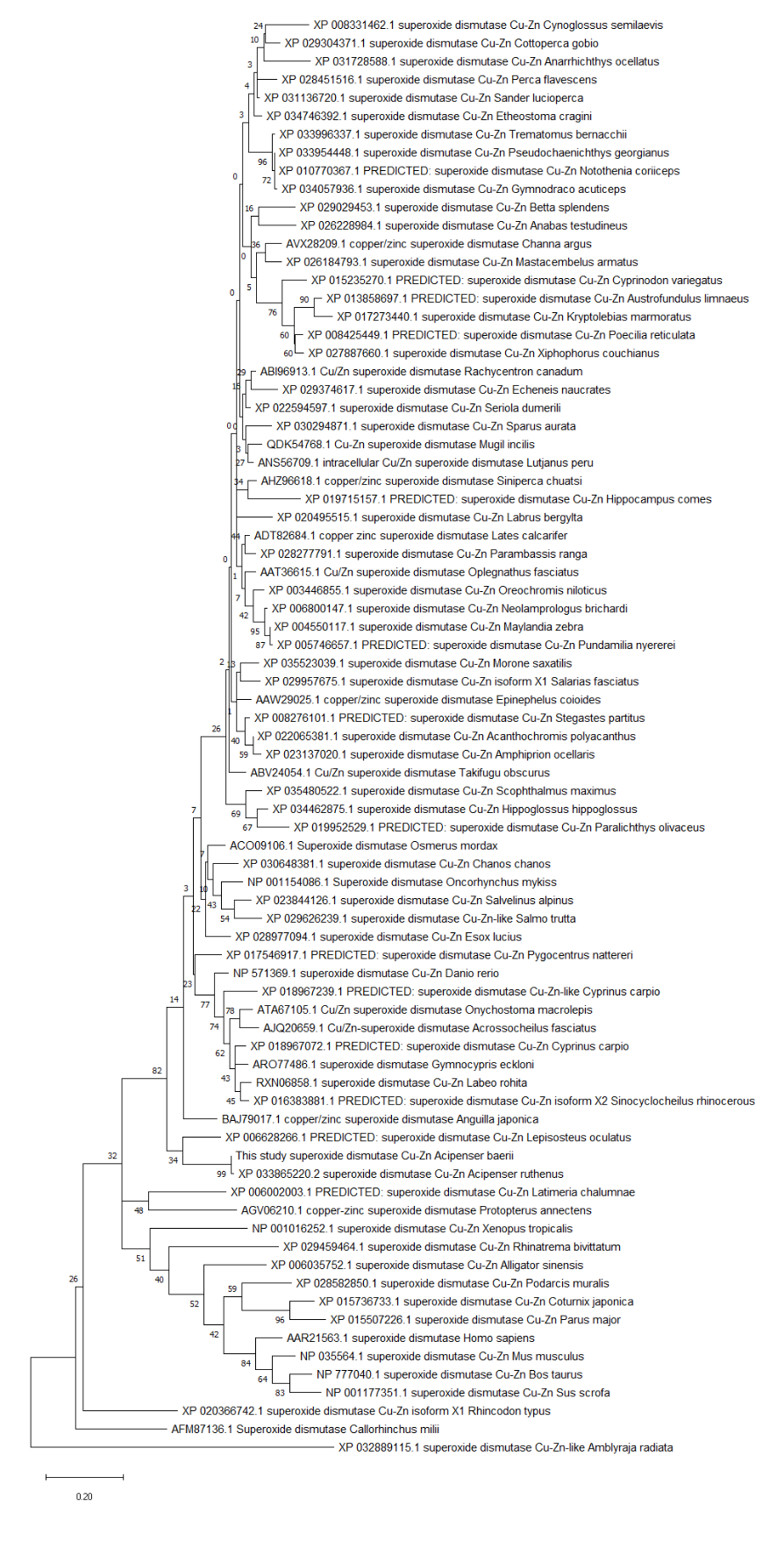


**Figure S6**. Phylogenetic tree analysis of SOD1 proteins in jawed vertebrates by ML method. The evolutionary history was inferred by using the determined best-fit evolutionary model (WAG+G). The tree with the highest log likelihood (-4996.61) is shown. The percentage of trees in which the associated taxa clustered together is shown next to the branches. Initial tree(s) for the heuristic search were obtained automatically by applying NJ and BioNJ algorithms to a matrix of pairwise distances estimated using the JTT model, and then selecting the topology with superior log likelihood value. A discrete Gamma distribution was used to model evolutionary rate differences among sites (5 categories (+*G*, parameter = 0.4303)). This analysis involved 79 amino acid sequences. All positions containing gaps and missing data were eliminated (complete deletion option). There were a total of 142 positions in the final dataset. The scale bar at the bottom of the tree represents amino acid substitution per site.


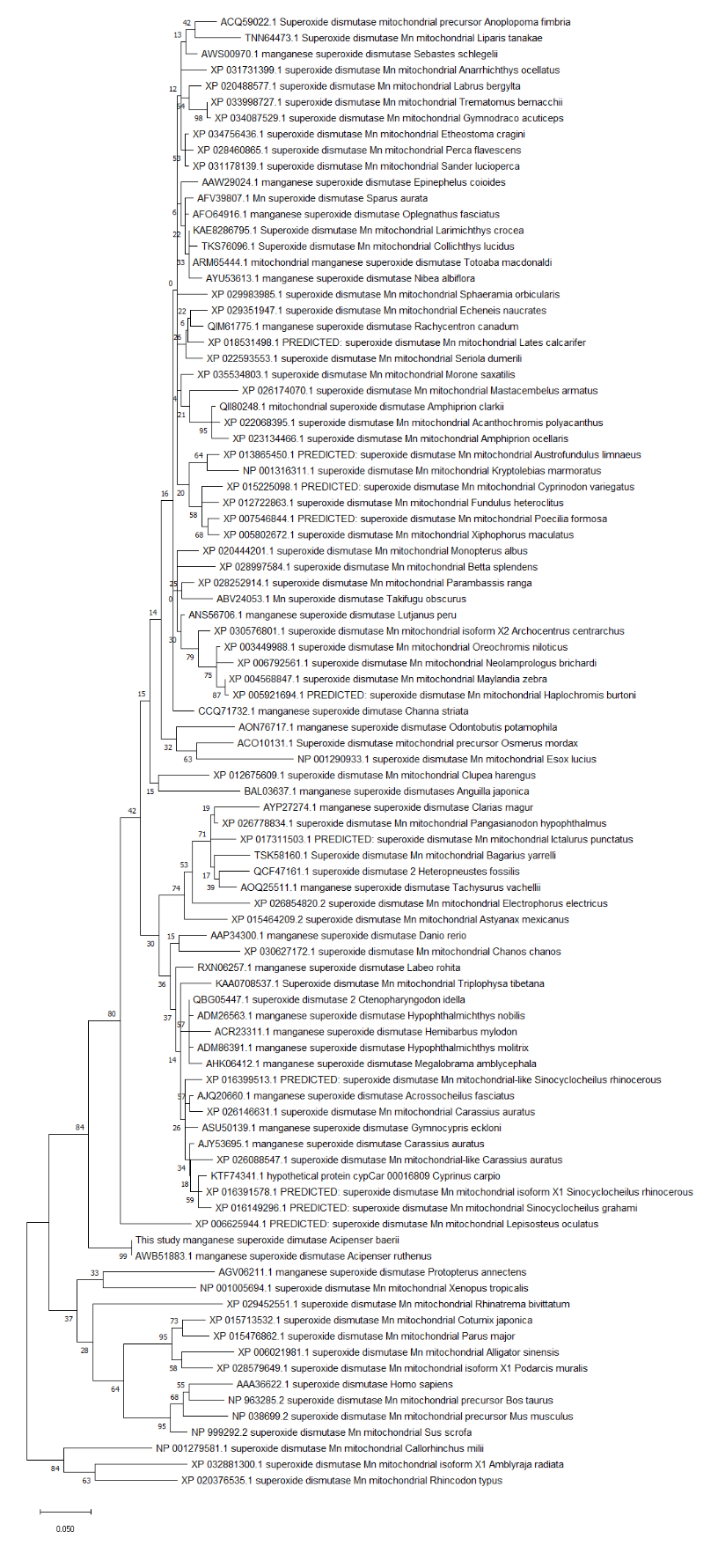


**Figure S7**. Phylogenetic tree for SOD2 proteins in jawed fishes by ML method. The evolutionary history was inferred by using determined best-fit evolutionary model (JTT+G). The tree with the highest log likelihood (-4610.32) is shown. The percentage of trees in which the associated taxa clustered together is shown next to the branches. Initial tree(s) for the heuristic search were obtained automatically by applying NJ and BioNJ algorithms to a matrix of pairwise distances estimated using the JTT model, and then selecting the topology with superior log likelihood value. A discrete Gamma distribution was used to model evolutionary rate differences among sites (5 categories (+*G*, parameter = 0.3682)). This analysis involved 92 amino acid sequences. All positions containing gaps and missing data were eliminated (complete deletion option). The scale bar at the bottom of the tree represents amino acid substitution per site.


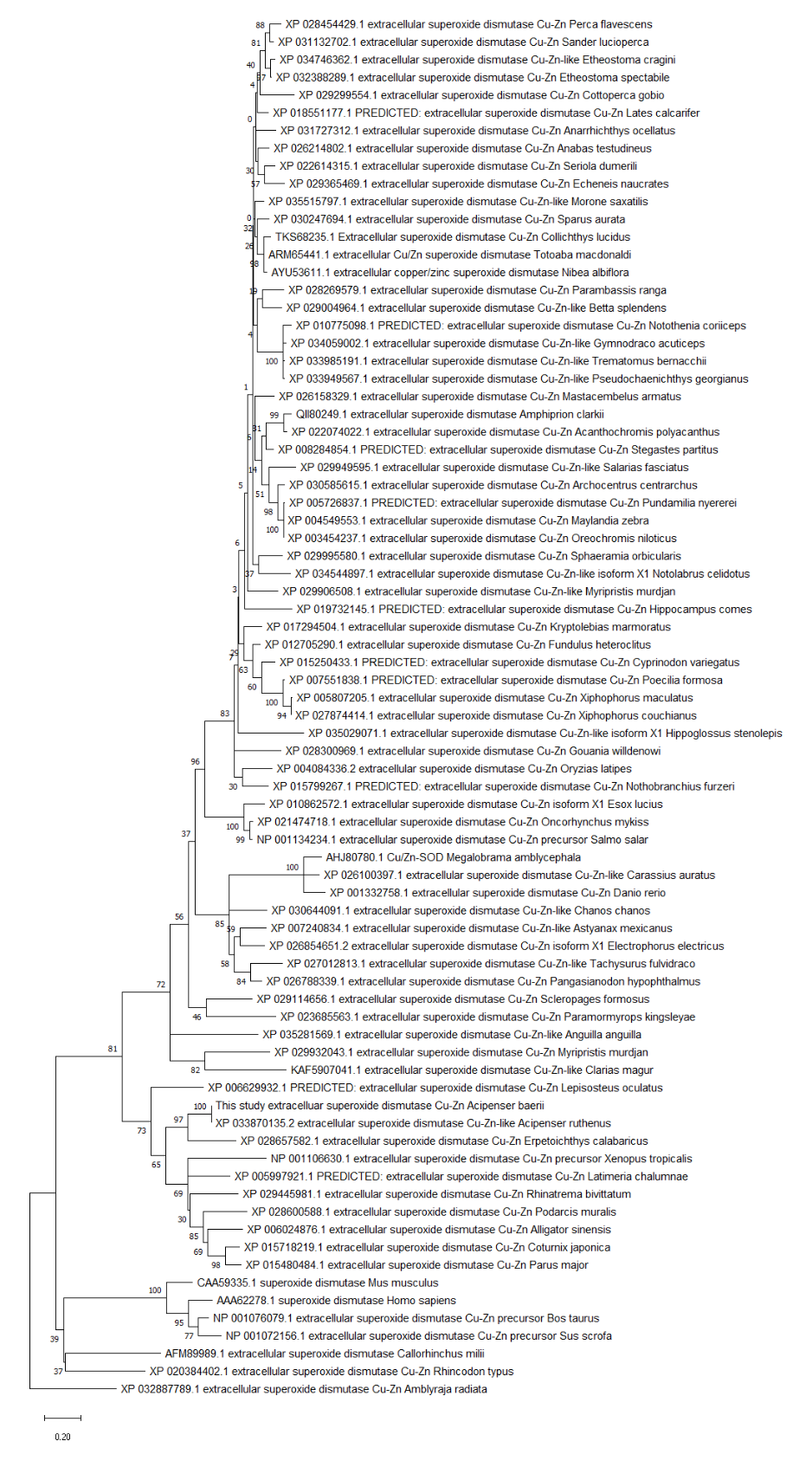


**(a)**

Continued Figure S8.


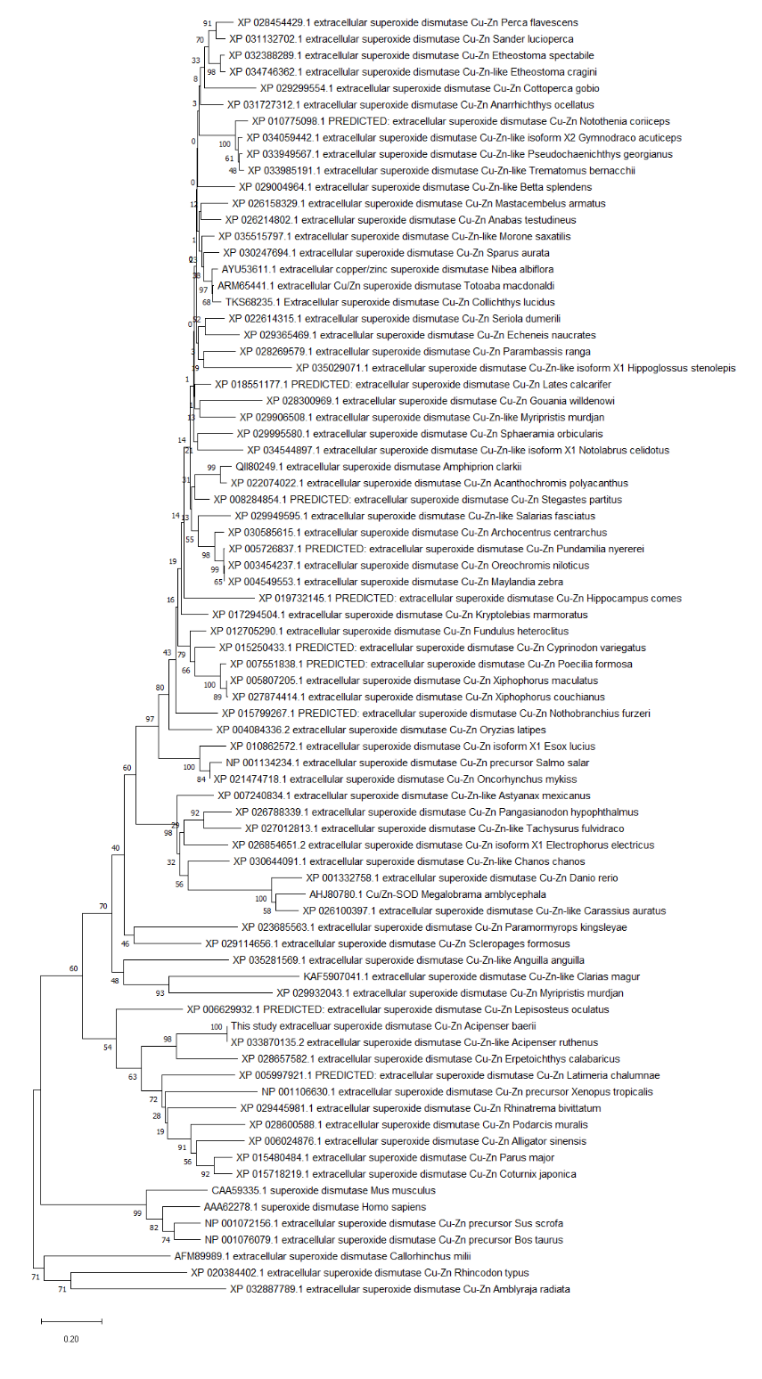


**(b)**

**Figure S8**. Phylogenetic tress of SOD3 proteins in jawed fishes by ML and NJ method. (a) The evolutionary history was inferred by using the determined best-fit evolutionary model (JTT+G+I). The tree with the highest log likelihood (-8848.61) is shown. The percentage of trees in which the associated taxa clustered together is shown next to the branches. Initial tree(s) for the heuristic search were obtained automatically by applying NJ and BioNJ algorithms to a matrix of pairwise distances estimated using the JTT model, and then selecting the topology with superior log likelihood value. A discrete Gamma distribution was used to model evolutionary rate differences among sites (5 categories (+*G*, parameter = 1.0834)). The rate variation model allowed for some sites to be evolutionarily invariable ([+*I*], 7.81% sites). This analysis involved 78 amino acid sequences. All positions containing gaps and missing data were eliminated (complete deletion option). There were a total of 160 positions in the final dataset. The scale bar at the bottom of the tree represents amino acid substitution per site. (b) The evolutionary history was inferred using the Neighbor-Joining method [1]. The optimal tree with the sum of branch length = 12.95677752 is shown. The percentage of replicate trees in which the associated taxa clustered together in the bootstrap test (1000 replicates) are shown next to the branches. The evolutionary distances were computed using the JTT matrix-based method and are in the units of the number of amino acid substitutions per site. The rate variation among sites was modeled with a gamma distribution (shape parameter = 1.08). This analysis involved 78 amino acid sequences. All positions containing gaps and missing data were eliminated (complete deletion option).


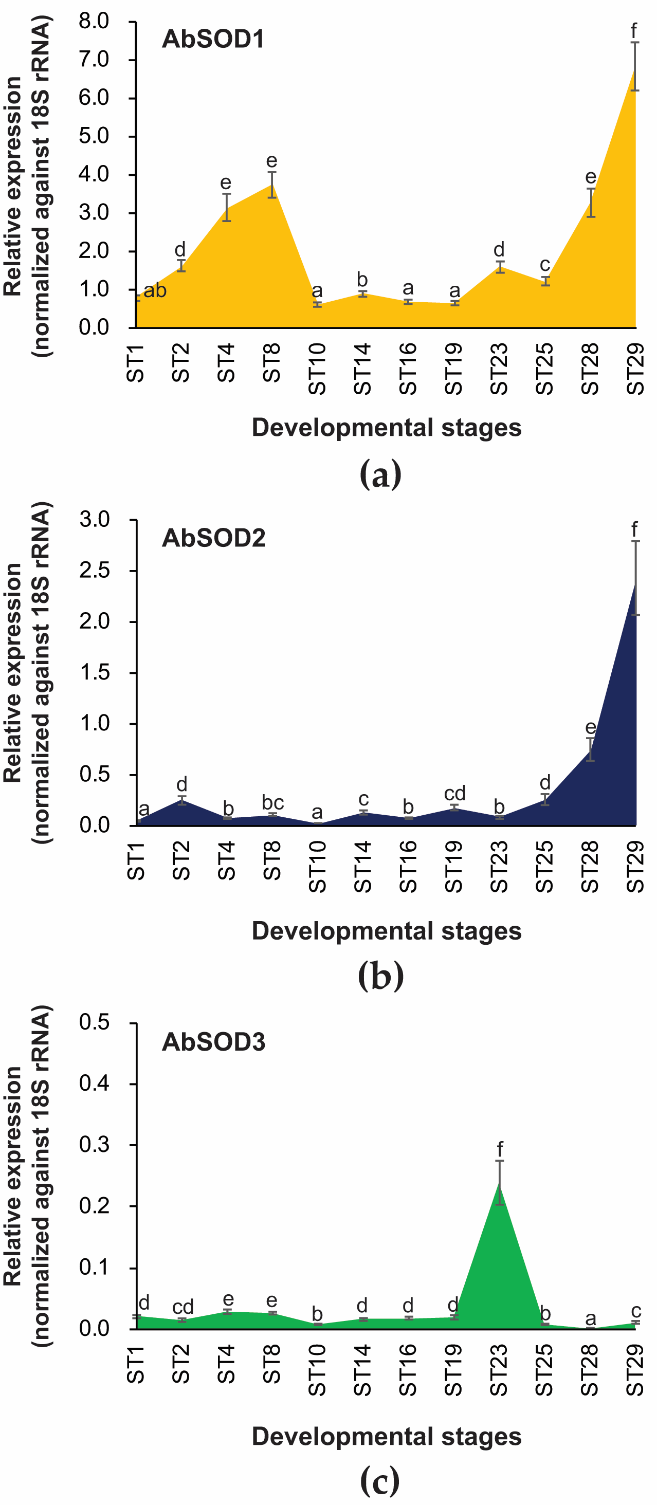


**Figure S9**. Statistical test results for embryonic expression of SOD isoforms. (**a**) AbSOD1, (**b**) AbSOD2, (**c**) AbSOD3.
