# Supplementary material for: Superoxide Dismutase Multigene Family from a Primitive Chondrostean Sturgeon, Acipenser baerii: Molecular Characterization, Evolution, and Antioxidant Defense during Development and Pathogen Infection
Source: Antioxidants (Basel). 2021 Feb 3;10(2):232. doi: 10.3390/antiox10020232 (PMC7913737; doi:10.3390/antiox10020232)
Supplement: Supplementary file 1 [file antioxidants-10-00232-s001.zip › Table S1.docx]

Table S1. Oligonucleotide primers used in this study

| Gene | Primer name | Sequence (5´-3´) | Purpose |
| --- | --- | --- | --- |
| AbSOD1 | AbSOD1-5Race | TGCCCTTCAGAACGCAAACA | RACE |
|  | AbSOD1-3Race | GCCTGCGGAGTAATTGGAAT |  |
|  | AbSOD1-FW | TGTGCGGAGCTGAAGATTGA | Isolation of full ORF  (amplicon = 206 bp) |
|  | AbSOD1-RV | ATGCTGCAACATTTCAGGGC |  |
|  | AbSOD1-qF | GTCGAACTATGGTGATCCAC | RT-qPCR assay |
|  | AbSOD1-qR | GGGCTTTGATATTGCCCTAC |  |
| AbSOD2 | AbSOD2-5Race | AGCAACACAGCTTAGAGCTG | RACE |
|  | AbSOD2-3Race | AGCGATCTGGAACGTGGTCA |  |
|  | AbSOD2-FW | CGCTGTGTAAATGAGAGTCTG | Isolation of full ORF |
|  | AbSOD2-RV | CAGAGTCGTGAGCATACATTG |  |
|  | AbSOD2-qF | TCACCCCTCTGCTTGGAATT | RT-qPCR assay  (amplicon = 218 bp) |
|  | AbSOD2-qR | ATGTTCACAGCCGCAGTTGT |  |
| AbSOD3 | AbSOD3-5Race | GTTCTCCTCAGATGTTGGAG | RACE |
|  | AbSOD3-3Race-1 | GAGGTTAGCTTGCTGTGTCA |  |
|  | AbSOD3-3Race-2 | GACATTCCCATCCACAGTAC |  |
|  | AbSOD3-FW | TGATGAGGCTGCAGCGTATT | Isolation of full ORF |
|  | AbSOD3-RV | CACTACTGCAACTCCTGTTG |  |
|  | AbSOD3-qF | GCCAGTCTACTGAATGGCAA | RT-qPCR assay  (amplicon = 214 bp) |
|  | AbSOD3-qR | AAAACGTTGCACTGGAGCCA |  |
| 18S rRNA | Ab18S-q1F | ATACAGGACTCTTTCGAGGC | RT-qPCR assay (normalization control; amplicon = 248 bp) |
|  | Ab18S-q1R | CTCAGTTAAGAGCATCGAGG |  |
